# Plasma proteomic analysis of autoimmune hepatitis in an improved AIH mouse model

**DOI:** 10.1186/s12967-019-02180-3

**Published:** 2020-01-06

**Authors:** Han Wang, Wei Yan, Zuohua Feng, Yuan Gao, Liu Zhang, Xinxia Feng, Dean Tian

**Affiliations:** 1 Department of Gastroenterology, Tongji Hospital, Tongji Medical College, Huazhong University of Science and Technology, Jiefang Avenue 1095, Wuhan, 430030 People’s Republic of China; 2 Department of Biochemistry and Molecular Biology, School of Basic Medicine, Tongji Medical College, Huazhong University of Science and Technology, Wuhan, 430030 People’s Republic of China

**Keywords:** Autoimmune hepatitis, Mouse model, Proteome, KEGG, SAA1

## Abstract

**Background:**

The prevalence of autoimmune hepatitis (AIH) is increasing, and its early clinical diagnosis is difficult. The pathogenesis of AIH remains unclear, and AIH-related studies are largely limited because of lack of suitable mouse models.

**Methods:**

To obtain a good tool for research on AIH, we first established an improved immune-mediated mouse model that can mimic the pathological process of AIH as in the human body, through repeated injections of human cytochrome P450 2D6 (*CYP2D6*) plasmid. Next, a proteomic analysis based on isobaric tag (IBT) technology was performed to detect the differentially expressed proteins (DEPs), and related biological functions and pathways in the plasma of AIH and normal mice. Finally, we performed enzyme-linked immunosorbent assay (ELISA) to further confirm the most abundant DEP in the plasma of patients with AIH.

**Results:**

Autoantibodies and the characteristic pathology of AIH were observed in our mouse model. Inflammatory infiltration also increased in the livers of AIH mice over time and plateaued by day 42 post the first injection. Chronic hepatitis was most severe on day 35 with the development of fibrosis as well, and the plasma of AIH mice were collected for proteomic analysis. A total of 176 DEPs were found in this experiment, of which 148 DEPs were up-regulated and 28 DEPs were down-regulated. Thirty significant Kyoto Encyclopedia of Genes and Genomes (KEGG) pathways (P < 0.05) were detected. Arginine biosynthesis was found to be the most significant pathway involved in the AIH process. During the Gene Ontology (GO) analysis, most DEPs were found to be involved in the binding, cellular, and metabolic processes. Using ELISA, the most overexpressed DEP, serum amyloid A 1 (SAA1), was confirmed to be increased specifically in the plasma of patients with AIH compared to other chronic hepatitis. Different plasma levels of SAA1 were also found related to different grades of inflammation and stages of fibrosis in the liver of patients with AIH.

**Conclusions:**

Our study is the first to describe the proteomics analysis of a true sense of AIH mouse model, which is beneficial for a better understanding of AIH pathogenesis and identifying potential biomarkers for its clinical diagnosis.

## Background

Autoimmune hepatitis (AIH) is a relatively rare inflammatory liver disease characterized by liver immune tolerance failure leading to the destruction of hepatic parenchyma. The prevalence of AIH has increased in recent years [1], and there is no effective treatment for AIH, except using immunosuppressive agents [2]. The precise etiology and the pathogenesis of AIH are still largely unknown because of lack of a reliable animal model to study this disease [3]. Concanavalin A (ConA) may induce a typical T cell-mediated hepatitis in mice characterized by immune cell infiltration and severe liver damage [4]. Many studies related to the pathophysiology of immunologically mediated hepatic disorders such as autoimmune chronic active hepatitis were based on this mouse model [5–7]. Mice developed severe liver injury, which was assessed through transaminase release within 8 h when an intravenous dose > 1.5 mg/kg ConA was given, and only the liver was affected [8]. However, ConA-induced hepatitis mouse model may not fully mimic AIH in the human body because the acute liver injury usually disappears after 48 h. Moreover, autoantibody production, liver fibrosis, and the characteristic pathology of AIH have not been observed in this mouse model.

Over the past decades, several researchers have tried to establish appropriate animal models, but a widely accepted AIH mouse model has not yet been recognized [4, 9, 10]. AIH is divided into two main types based on the serological autoantibody profile: type-1 AIH and type-2 AIH [2]. Type-1 AIH is defined by positivity to antinuclear antibodies (ANA) and/or anti-smooth muscle antibody (SMA), while type-2 AIH is characterized by the presence of anti-liver-kidney microsomal 1 (LKM-1) antibody [11]. Cytochrome P450 2D6 (CYP2D6) is a clearly recognized human autoantigen in type-2 AIH and it is the antigenic target of anti-LKM-1 antibody [12]. Christen et al. were the first to infect mice with an adenovirus expressing human *CYP2D6* (Ad-*2D6*) to establish the AIH mouse model [13]. Moreover, the human leukocyte antigen (HLA)-DR3 and -DR4 alleles have been known to be strongly linked to both type-1 and type-2 AIH; hence, related studies on AIH mouse models have also been reported [12, 14].

Early diagnosis of AIH in patients is important but may be challenging in clinical practice. At present, the criterion of the International Autoimmune Hepatitis Group (IAIHG) is used for AIH diagnosis [15]. However, the diagnostic criterion is a scoring system, and the accurate diagnosis usually requires the results of liver biopsy. Autoantibodies play an important role in AIH diagnosis, but do not show clinical specificity. They may not be present in several special patients with AIH but can be detected in other autoimmune diseases [16]. As AIH is a systemic disease and the blood sample is much easier to obtain from the human body, identifying new biomarkers in the serum/plasma is an urgent requirement for early clinical diagnosis of AIH. At present, a limited number of studies [17–19] has investigated the potential biomarkers for AIH, partly because of the difficulty in AIH diagnosis or lack of an animal model for this field of research.

Unlike the one-time Ad-*CYP2D6* (*CYP2D6* gene incorporated into adenovirus) infection determined by Christen et al., in the present study, we established, for the first time, a mouse model to mimic constantly the pathological process of AIH in vivo by combining the initial one-time adenovirus infection and repeated injections of human *CYP2D6* plasmid. This is an improved and novel method of establishing an AIH mouse model. Chronic inflammation, liver fibrosis, autoantibodies, and the characteristic pathology of AIH were observed in the mouse, suggesting that our mouse model could almost accurately mimic the pathogenesis of AIH in the human body. We also compared the autoantibodies observed in this mouse model with those in patients suffering from autoimmune liver diseases and we found that the autoantibodies in our mouse model were similar to those in type 2 patients with AIH. Then, we utilized isobaric tag (IBT) technology, which is an optimized analytical method based on IBTs for isobaric tags for relative and absolute quantitation (iTRAQ), for quantitative determination of proteins in the plasma of AIH mice and normal mice. Moreover, the biological and metabolic processes and the related pathways in the AIH mouse models have also been explored. According to the IBT results, the levels of serum amyloid A (SAA) proteins increased most significantly in the AIH mouse plasma. The function of SAA proteins, which act as cytokine-like proteins, has recently been recognized in cell–cell communication, as well as in feedback in several inflammatory processes [20]. Moreover, SAA1, which is the most abundant DEP in our study, has been proven to aggravate T cell-mediated hepatitis by inducing chemokines in a ConA mouse model [21]. However, little research has been undertaken on the expression of SAA family proteins in the plasma of patients with AIH.

Overall, our work described an improved and steady AIH mouse model that mimics disease conditions in patients with type-2 AIH, which could be a good tool for this research field. Further, we analyzed the DEPs and the biological pathways in this model using IBT, which may provide us with a better understanding of AIH.

## Methods

### AIH mouse model

**Fig. 1:**
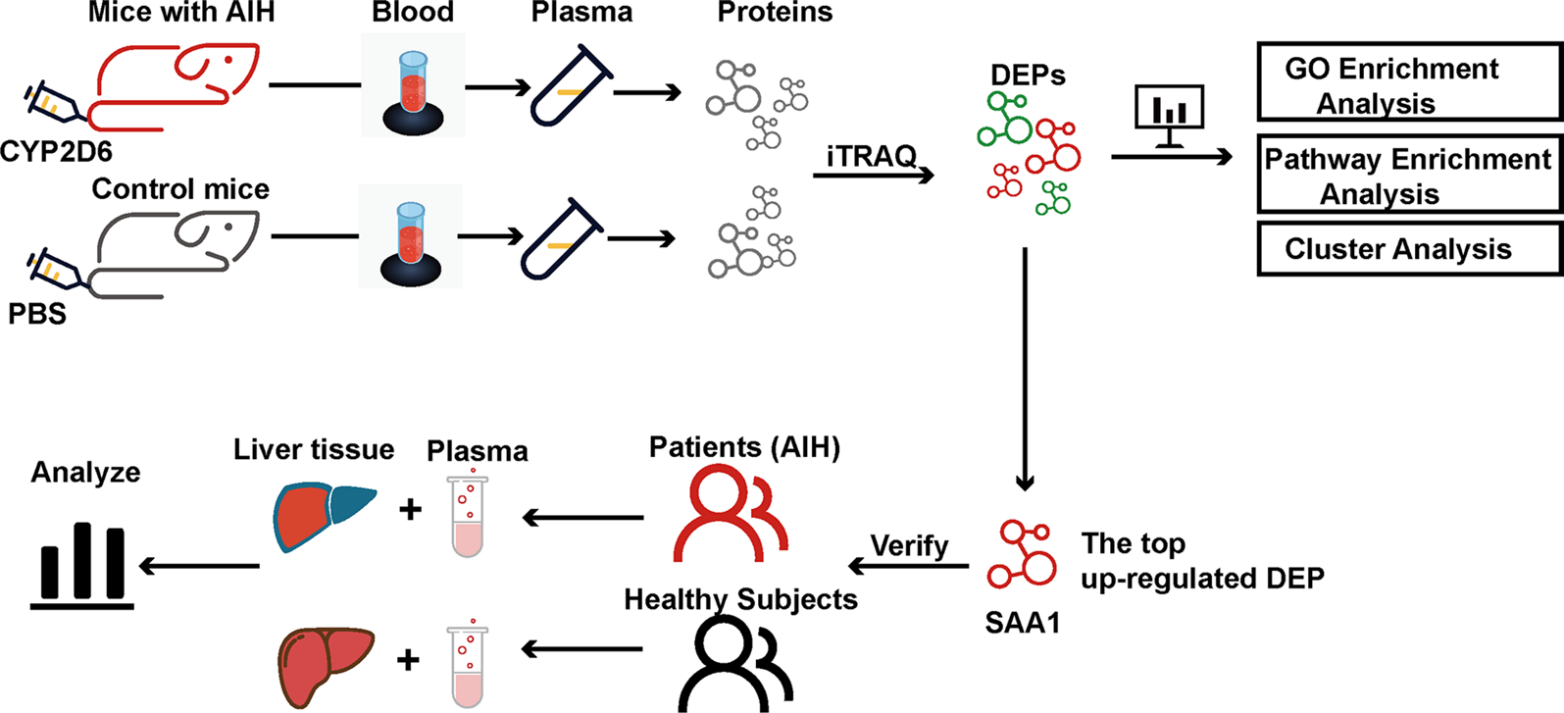
The schematic diagram of the experiment design. *CYP2D6* cytochrome P450 2D6, *AIH* autoimmune hepatitis, *DEPs* differentially expressed proteins, *iTRAQ* isobaric tags for relative and absolute quantitation, *SAA1* serum amyloid A 1, *GO* gene ontology

Specific pathogen-free (SPF) male C57BL/6 mice (6–8 weeks old, 18–20 g) were purchased from Beijing Vital River Laboratory Animal Technology Co., Ltd. (Beijing, China). The mice were housed in an SPF environment with an alternating 12 h light/dark cycle at 24 ± 2 °C and relative humidity of 55–60%. A plasmid expressing human *CYP2D6* (p*CYP2D6*) was constructed. Mice were first infected with adenovirus through tail vein injection on day 0 to promote the induction of AIH using human *CYP2D6*. Next we injected p*CYP2D6* plasmid at day 1, 4, 9, and 13 via a rapid tail vein injection (50 μg per injection) to transfect human *CYP2D6* into the mice livers using the hydrodynamic-based liver-targeted gene delivery technique [22]. Plasmid injection could be performed once before adenovirus injection to enhance immunogenicity (at day − 1). The detailed protocol is shown in Additional file 1: Figure S1. Mice were sacrificed on days 14, 28, 35, and 42 after the first injection. Mice blood and liver tissues were collected for hematoxylin and eosin (H&E) staining, Sirius red staining, immunofluorescence (IF) analysis, immunohistochemistry (IHC), western blot analysis, and quantitative polymerase chain reaction (qPCR). Mice plasma collected from the angular vein on day 35 were used for IBT analysis. The following study setup design is shown in a schematic diagram (Fig. 1).

### Mouse plasma preparation and high abundance protein depletion

Venous blood from five AIH mice and five normal mice was collected from the angular vein using the anticoagulant tubes, which were pretreated with citrate-dextrose solution (Sigma-Aldrich, St. Louis, MO USA) and then centrifuged at 500*g* for 10 min to obtain the supernatant (plasma). To obtain and concentrate as much of the low-abundant proteins as possible, the samples were equalized using the ProteoMiner Protein Enrichment Kit (Bio-rad laboratories, Hercules, CA, USA), according to the manufacturer’s instructions. Each column was loaded with the samples, which were first passed through a 0.22-μm-filter. No bead agglomeration was observed. The proteins were desorbed using a two-step elution process. First, the beads were treated twice with 100 μL of the kit elution reagent (4 M urea, 1% (w/v) CHAPS, 5% (v/v) acetic acid) for 15 min. Then, 100 μL of 6 M guanidine-HCl (pH 6.0) was added twice, followed by incubation for 15 min. Finally, four elution fractions from each column were pooled and stored at − 80 °C for further analysis.

### Protein quantitation and digestion

Proteins were quantified with Bradford assay, and then they were double verified by SDS-PAGE. For digestion, the protein solution (100 μg) with 8 M urea was diluted 4 times with 100 mM TEAB. Trypsin Gold (Promega, Madison, WI, USA) was used to digest the proteins with the ratio of protein: trypsin = 40: 1 at 37 °C overnight. After trypsin digestion, peptides were desalted with a Strata X C18 column (Phenomenex) and vacuum-dried according to the manufacturer’s protocol.

### Peptide labeling and fractionation

The peptides were dissolved in 25 μL of 0.2 M TEAB, and were mixed thoroughly. After being kept at room temperature, the IBT labeling reagents were dissolved in 80 μL of isopropanol by vortexing. Next, we combined these mixtures with the proper samples. Peptide labeling was performed using the IBT reagent kit, according to the manufacturer’s protocol. The peptides labeled with different reagents were combined and vacuum-dried for further use. For peptide fractionation, we used a Shimadzu LC-20AB HPLC pump system coupled with a high pH RP column. The peptides were resuspended in buffer A (5% ACN, 95% H_2_O, adjusted pH to 9.8 with ammonia) up to 2 mL volume and loaded onto a column containing 5-μm particles (Phenomenex). The peptides were then separated at a flow rate of 1 mL/min with a gradient of 5% buffer B (5% H_2_O, 95% ACN, pH adjusted to 9.8 with ammonia) for 10 min, 5–35% buffer B for 40 min, and 35–95% buffer B for 1 min. The whole system was maintained in 95% buffer B for 3 min and within 1 min, the concentration of buffer B decreased to 5%. Next, they were balanced with 5% buffer B for 10 min. Elution was monitored by detecting absorbance at 214 nm, and fractions were collected every 1 min. The eluted peptides were pooled as 20 fractions and vacuum-dried.

### HPLC and mass spectrometry analysis

Each fraction was resuspended in buffer A (2% ACN and 0.1% FA in water) and centrifuged at 20,000*g* for 10 min. The supernatant was loaded onto a C18 trap column by the autosampler at a flow rate of 5 μL/min for 8 min using an LC-20AD nano-HPLC instrument (Shimadzu, Kyoto, Japan). The peptides separated by the nano-HPLC were analyzed using the tandem mass spectrometry Q EXACTIVE HF system (Thermo Fisher Scientific, San Jose, CA) for DDA (data-dependent acquisition) by nano-electrospray ionization. The parameters for MS analysis are as follows: electrospray voltage, 1.6 kV; precursor scan range, 350–1500 m/z at a resolution of 35,000 in Orbitrap; MS/MS fragment scan range, > 100 m/z at a resolution of 35,000 in HCD mode; normalized collision energy setting, 30%; dynamic exclusion time, 15 s; automatic gain control (AGC) for full MS target and MS2 target, 3e6 and 1e5, respectively; and the number of MS/MS scans following one MS scan, 20 most abundant precursor ions above a threshold ion count of 20,000.

### Protein quantification

We used an automated software called IQuant [23] for quantitatively analyzing the labeled peptides with IBTs. Based on a simple principle (the parsimony principle), the identified peptide sequences were assembled into a set of confident proteins. To control the rate of false-positive results at the protein level, 1% of protein FDR, which is based on the picked protein FDR strategy [24], was also set as the criteria for protein identification (protein-level FDR ≤ 0.01). The protein quantification process includes the following steps: protein identification, tag impurity correction, data normalization, missing value imputation, protein ratio calculation, statistical analysis, and result presentation.

### Bioinformatics analysis

The raw MS/MS data were converted to the MGF format using the corresponding tool, and the exported MGF files were searched using the local Mascot server against the relevant database. In addition, quality control (QC) was performed to determine if a reanalysis was needed. IQuant was utilized for the quantification of proteins. All proteins with FDR of less than 1% were subjected to downstream analyses, including Gene Ontology (GO) and Kyoto Encyclopedia of Genes and Genomes (KEGG) pathway analyses. We further performed a deeper analysis based on differentially expressed proteins (DEPs), including cluster analysis, GO enrichment analysis and KEGG pathway enrichment analysis. We also analyzed the interaction between the significant KEGG pathways and performed the protein–protein interaction (PPI) analysis.

### Plasma and liver tissues of patients

The collection of samples was approved by the local ethical committee and the institutional review board of Huazhong University of Science and Technology. Each patient provided written informed consent. The diagnoses of AIH, primary biliary cirrhosis (PBC), hepatitis B, and hepatitis C were made at Tongji Hospital and the selected patients had no history of immunosuppressant use. Liver biopsy was conducted on all selected patients with AIH, and the score reached seven points according to the IAIHG diagnostic criteria. The grade of inflammation and stage of fibrosis in the liver of patients with AIH was diagnosed by the pathologist in Tongji Hospital using the modified Scheuer histologic scoring system [25]. Venous blood from 30 patients with AIH, 30 patients with hepatitis B, 30 patients with hepatitis C, and 30 healthy people were collected for enzyme-linked immunosorbent assay (ELISA), while plasma samples from three patients with PBC, three patients with type-1 AIH, and three patients with type-2 AIH were used for immunofluorescence analysis. The patient blood samples (3 mL) were collected using anticoagulant tube and placed on ice for 20 min. Then the blood was centrifuged at 400*g* for 20 min to obtain the plasma, which was stored at − 80 °C until ELISA analysis. Liver tissues were collected from patients with AIH who needed pathological examination. The control liver tissues were collected from patients who underwent benign liver tumor resection. Tissues from liver biopsy were fixed with 4% paraformaldehyde for 48 h and were then embedded in paraffin and cut to obtain 5 μm thick samples. The tissue slides were used for immunohistochemical staining.

### Western blot analysis and Real-time qPCR

#### Western blot

Cell extracts and liver tissues were digested in 1× RIPA buffer containing phosphatase inhibitor, cocktail, and PMSF (Boster, Wuhan, China). Tissue protein (30 μg) was separated in an SDS polyacrylamide gel and was transferred to PVDF membranes. The blotted membranes were blocked with 5% BSA in TBST for 1 h at room temperature and they were incubated overnight at 4 °C on shaking tables with the following primary antibodies: anti-CYP2D6 (1:1000; sc-130366, Santa Cruz Biotechnology, CA, USA) and anti-GAPDH (1:2000; GB11002, Promoter Biotechnology, Wuhan China). The membranes were washed and incubated with horseradish peroxidase-conjugated secondary antibody (Promoter Biotechnology Wuhan China) for 1 h at room temperature. The expression of the antibody-linked protein was determined by enhanced chemiluminescence using an ECL assay kit (Boster, Wuhan, China).

#### Real-time quantitative PCR

Total RNA was isolated and extracted from the liver tissues using TRIzol reagent (Takara, Otsu, Japan). It was transcribed into cDNA using the reverse transcription kit (RR036A, Takara, Otsu, Japan) according to the manufacturer’s protocols. The relative mRNA levels were evaluated by quantitative PCR using Maxima SYBR Green qPCR Master Mix (Takara, Otsu, Japan). The primers used for real-time quantitative PCR are listed in Additional file 2: Table S1.

### Histopathology, immunofluorescence and immunohistochemistry analysis

The entire left lobe of the mouse liver was cut and fixed in 4% paraformaldehyde for at least 24 h, embedded in paraffin and cut at a thickness of 5 μm. Following hydration in a decreasing ethanol gradient, all sections were deparaffinized and stained with Harris hematoxylin solution for 5 min at 37 °C. For immunofluorescence, frozen sections of liver tissue samples were blocked with 5% horse serum for 30 min at room temperature and incubated with primary anti-αSMA (1:100; 55135-1-AP, Proteintech, Wuhan, China), anti-CD4 (1:100; 11056-2-AP, Proteintech, Wuhan, China), and anti-CD8a (1:200; #98941, Cell Signaling Technology, Danvers, MA USA) overnight at 4 °C. Next, the samples were incubated with Alexa Fluor 488 conjugated and Cy3-conjugated goat anti-rabbit secondary antibodies (1:100; Promoter biotechnology, Wuhan, China) for 1 h at room temperature. The nucleus was stained with Hoechst 33258 (1:1000; 17520, AAT Bioquest, Sunnyvale CA, USA). Indirect immunofluorescence staining for the detection of antibodies was performed on rat liver sections. The plasma from AIH mice and patients were diluted 1:300 and used as the primary antibody and then detected using DyLight 488 conjugated goat anti-mouse IgG (1:100; A23210, Abbkine, Waltham, MA USA) and FITC-conjugated goat anti-human IgG (1:100; SA00003-12, Proteintech, Rosemont, IL USA). The detailed protocol has been described previously [10, 13]. The final sections were examined using a confocal laser scanning microscope.

For the detection of SAA1 expression in the human liver tissues by immunohistochemistry, the tissue sample slides were deparaffinized with dimethylbenzene, followed by gradient alcohol dehydration. Endogenous peroxidase was blocked by 3% hydrogen peroxide. Primary antibody against SAA1 (1:100; 16721-1-AP, Proteintech, Rosemont, IL USA) was incubated overnight at 4 °C. The secondary antibody was incubated for 1 h at room temperature and was developed using peroxidase-conjugated streptavidin and DAB. Finally, slides were counterstained with hematoxylin and analyzed under a microscope.

### Elisa analysis

The collected plasma of patients were used to detect the expression of SAA1 level by SAA1 Elisa kit (#ELH-SAA-1, RayBiotech, Peachtree Corners, GA USA) according to the corresponding manufacturer’s instructions. The specific antibodies in the mice plasma were detected using Elisa and the detailed protocol was described in a previous study [13].

### Statistical analysis for RT-qPCR, Western blot, and Elisa

Results are expressed as mean ± standard error and all experiments were performed in triplicate independently. One-way analysis of variance was performed to assess the significant differences, and post-test were conducted using Tukey’s multiple comparisons test or Dunnett’s multiple comparisons test if the P-value were significant. SAA1 levels in human plasma were compared using Mann–Whitney and Kruskal–Wallis nonparametric tests. Statistical analysis was performed with GraphPad Prism 5.0 (GraphPad Software, La Jolla, CA, USA) and P < 0.05 was considered to indicate a statistically significant difference.

## Results

### Chronic liver inflammation and pathological features of AIH in the mouse model

**Fig. 2:**
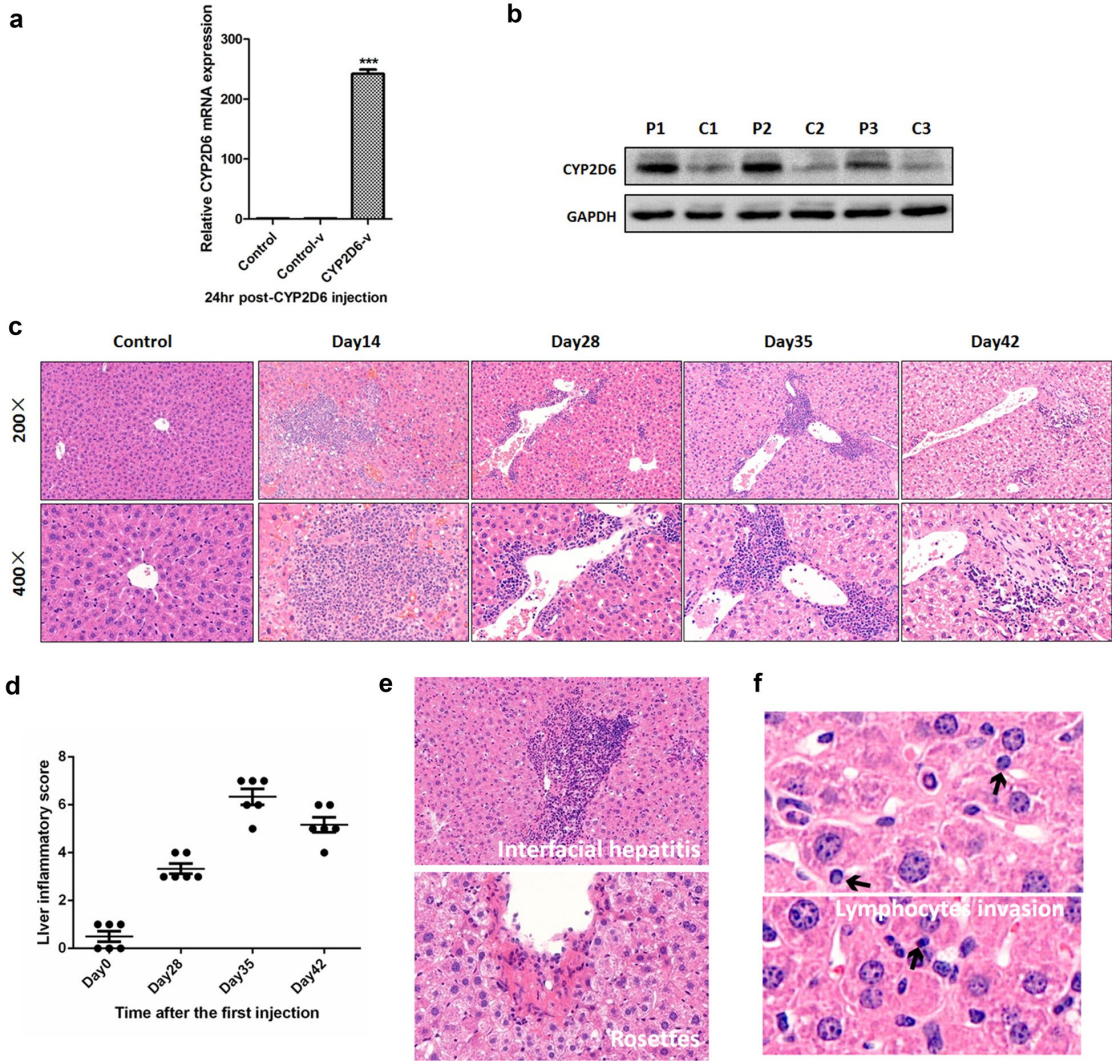
Chronic liver inflammation and pathological features of autoimmune hepatitis in the mouse model. **a** Real-time PCR was used to detect the transfection efficiency of the *CYP2D6* plasmid in the mouse livers (n = 6, ***P < 0.001 vs. Control). **b** The expression of CYP2D6 protein in the mouse liver was detected by western blotting (n = 3; P1–3 indicates the mice injected with *CYP2D6* plasmid and C1–3 indicates control mice injected with PBS). **c** C57 BL/6 mice were sacrificed at day 14, day 28, day 36, and day 42 post the first injection. The liver tissue was harvested and analyzed by H&E staining (n = 6, ×200 and ×400 magnification). Only one control group which injected PBS was shown in this figure. Other control groups (only injected with adenovirus or plasmid) were shown in Additional file 3: Figure S2. **d** Liver inflammatory score of the AIH mouse model at different days (based on the Knodell histological activity index, HAI, a hepatitis scoring system). **e** Interfacial hepatitis was observed in the mouse livers at day 36, and rosettes and lymphocytes invasion were also detected (**f**); the arrows indicate the lymphocytes

To detect the efficiency of p*CYP2D6* transfection in the mouse liver using hydrodynamic-based liver-targeted gene delivery technique, qRT-PCR (Fig. 2a) and western blotting analyses were used to detect the expression of CYP2D6 in the mouse liver (Fig. 2b). Mice were sacrificed on different days (day 14, day 28, day 35, and day 42) after the first adenovirus injection. The representative H&E staining of the livers from mice in different groups are shown in Fig. 2c; the control groups (mice receiving only adenovirus injection or only p*CYP2D6*) are described in Additional file 3: Figure S2. Inflammatory cells began to infiltrate the liver on day 14, and chronic lymphocytes appeared around the portal vein. Liver inflammation became most severe on day 35, with visible fibrosis. Then, hydropic degeneration of the liver cells was observed on day 42, while the infiltration by inflammatory cells became mild, but obvious fibrosis could be observed. The liver inflammatory score on different days was assessed by three doctors in the pathology department of the Tongji hospital using the Knodell histological activity index (Knodell, HAI), a hepatitis scoring system (Fig. 2d). Furthermore, characteristic pathological features of AIH were detected in the mouse livers on day 35, including interfacial hepatitis and rosettes (Fig. 2e). Invasive lymphocytes, a phenomenon whereby a lymphocyte stretches into a hepatocyte and disappears was also seen in the AIH mouse liver (Fig. 2f). The histological characteristics observed in our results support this mouse model as an adaptive model for studying AIH.

### The occurrence of liver fibrosis in the AIH mouse model

**Fig. 3:**
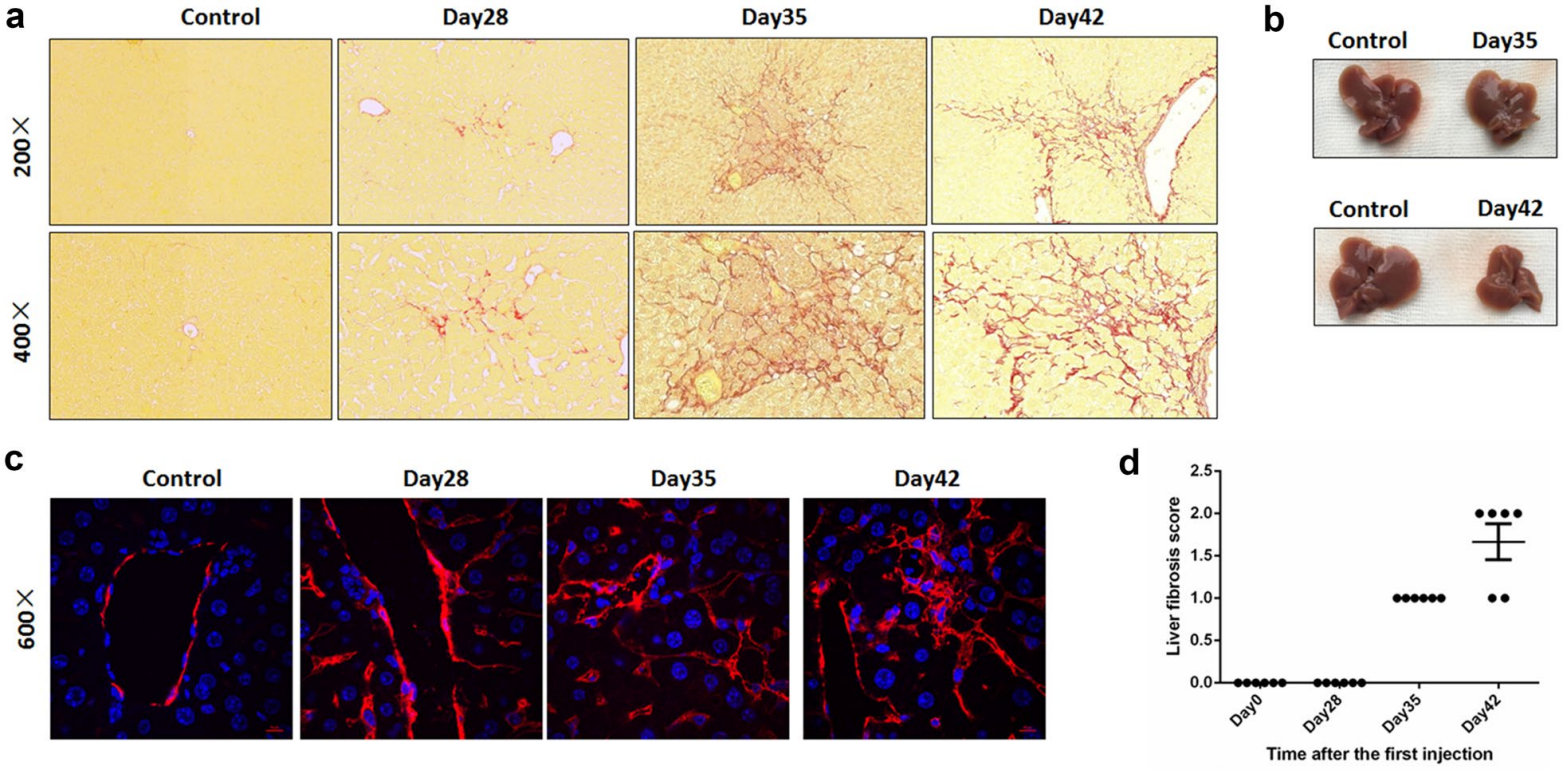
Occurrence of liver fibrosis in the AIH mouse model. **a** Mouse liver tissues were harvested at different days for Sirius red staining. Representative pictures are shown (n = 6, ×200 and ×400 magnification). **b** Gross appearance of the mouse liver at different days. **c** α-SMA (red) was detected by immunofluorescence staining (×600 magnification). Nuclei were stained with Hoechst 33258 (blue). **d** Liver fibrosis score of AIH mouse model at different days (based on the Knodell histological activity index, *HAI* a hepatitis scoring system)

Considering that fibrosis is one of the main features of terminal AIH, we used Sirius red staining to detect the fibrosis level on different days in the mouse liver (Fig. 3a). Mild liver fibrosis occurred on day 28, with more collagenous fibers growing around the vein in the liver. However, fibrosis became more severe on day35 and more collagenous fibers appeared in the liver parenchyma. The mouse livers became slightly smaller, based on the gross appearance (Fig. 3b). On day 42, the collagenous fibers connected different veins, and promoted the formation of pseudolobules in the mouse liver. At this time, the mouse liver showed a wizened, sclerotic gross appearance, and was much smaller than the normal liver as well (Fig. 3b). We also used immunofluorescence analysis to detect the expression of α-SMA in the mouse livers (Fig. 3c). α-smooth muscle actin (α-SMA) is normally expressed around the blood vessels, and its levels increased in the AIH mouse liver on day 28. It also expressed in the liver parenchyma, and its fluorescence intensity was strong, which provides evidence of fibrosis in the AIH mouse liver, from another point of view. Similarly, the liver fibrosis score was assessed using the HAI scoring system (Fig. 3d). Our data suggest that liver fibrosis in our mouse model is progressive and consistent with the features of patients with AIH.

### Autoantibody production and immune cells infiltration in the AIH mouse model

**Fig. 4:**
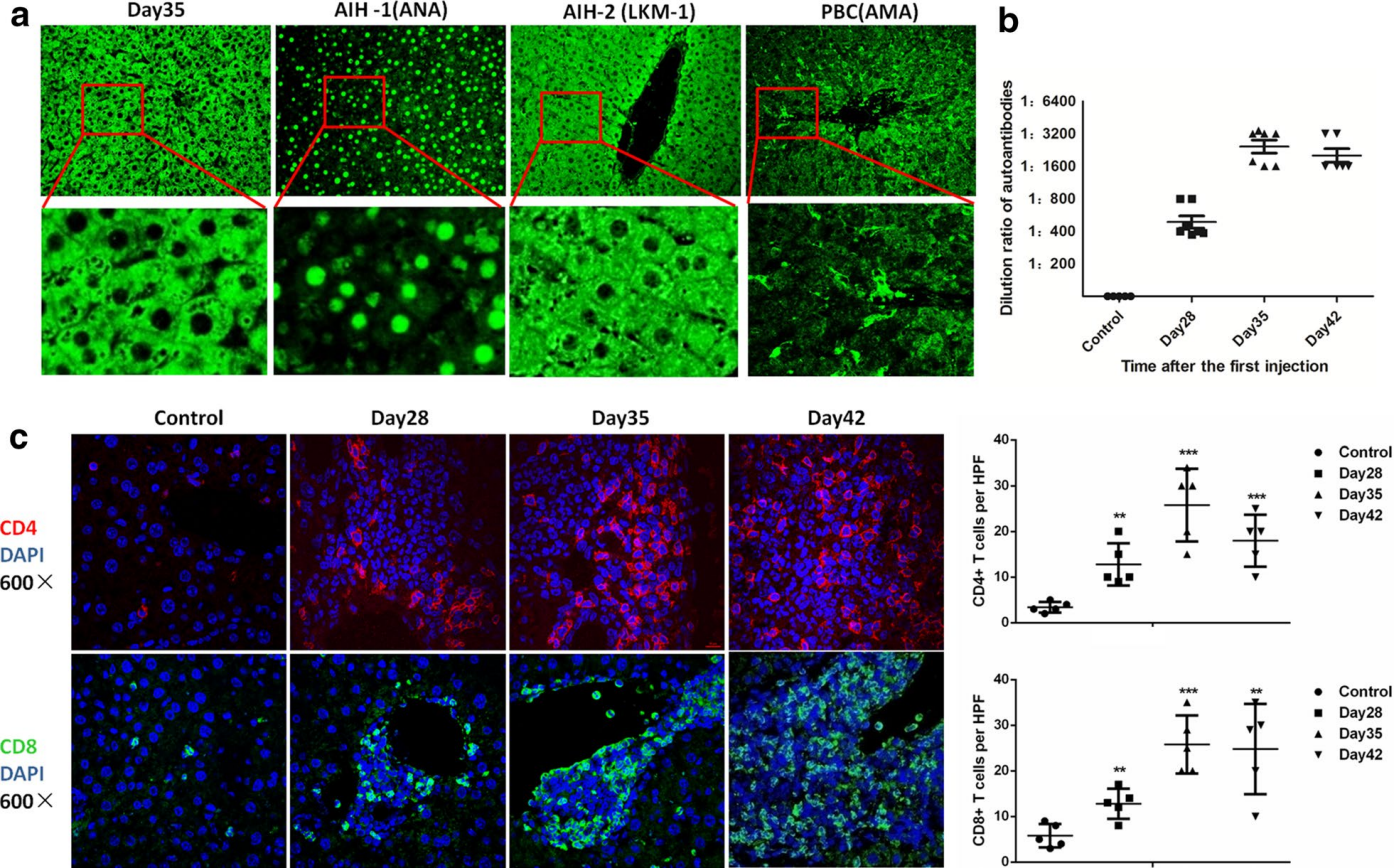
Autoantibody production and immune cell infiltration in the AIH mouse model. **a** Representative pictures of rat liver sections stained with plasma from AIH mice (primary antibody) at day 35, followed by Alexa Fluor 488-conjugated anti-mouse IgG (secondary antibody). The plasma from AIH-1 (type-1 autoimmune hepatitis) patients (antinuclear antibodies, ANAs), AIH-2 (type-2 autoimmune hepatitis) patients (anti-liver-kidney microsomal-1 (LKM-1) antibodies), and PBC patients (anti-mitochondrial antibodies, AMAs) were used to stain the same rat liver sections as the primary antibodies. FITC-conjugated anti-human IgG was used as the secondary antibody (n = 3, upper column: ×200 magnification, lower column: ×800 magnification). **b** The dilution ratio of autoantibodies in the plasma of AIH mouse after the first injection (n = 7). **c** CD4^+^ T cells and CD8^+^T cells were found to accumulate in the AIH mouse liver on different days after the first injection, with CD4 (red) and CD8 (green) as their marker respectively. Nuclei were stained with Hoechst (blue) (n = 5, ×600 magnification). **P < 0.01, ***P < 0.001 vs. the control group

The presence of autoantibodies in patient plasma is an essential element for the diagnosis of AIH. It is also an important criterion for the establishment of an accurate AIH mouse model. Therefore, we collected plasma from AIH mice and diluted it for use as the primary antibody. Simultaneously, plasma from patients with type-1 AIH, type-2 AIH, and PBC were also used for comparison (Fig. 4a). The autoantibodies in AIH mice were similar to the anti LKM-1 antibodies in patients with type-2 AIH. The anti-ANA in patients with type-1 AIH and anti-mitochondrial antibody (AMAs) in patients with PBC served as controls. We also performed ELISA to detect the titer of the autoantibodies; the results are shown in Fig. 4b. The concentration of autoantibodies reached its peak on day 35 and subsided on day42. Considering that AIH is a typical T-cell-mediated hepatitis, we further detected the infiltration of CD4^+^ T and CD8^+^T cells in the AIH mouse liver. As shown in the representative immunofluorescence images (Fig. 4c), an increasing number of CD4^+^ T cells and CD8^+^T cells infiltrated from the blood vessels on day 28. Moreover, they further increased in number and infiltrated the liver parenchyma on days 35 and 42. These findings indicate that there is indeed autoantibody production, and an increase in T cell infiltration in our mouse model. These results fully confirm that our improved AIH mouse model can mimic the pathogenesis and the characteristics of AIH in the human body.

### Venn and cluster results of IBT quantification among AIH and normal mice groups

**Fig. 5:**
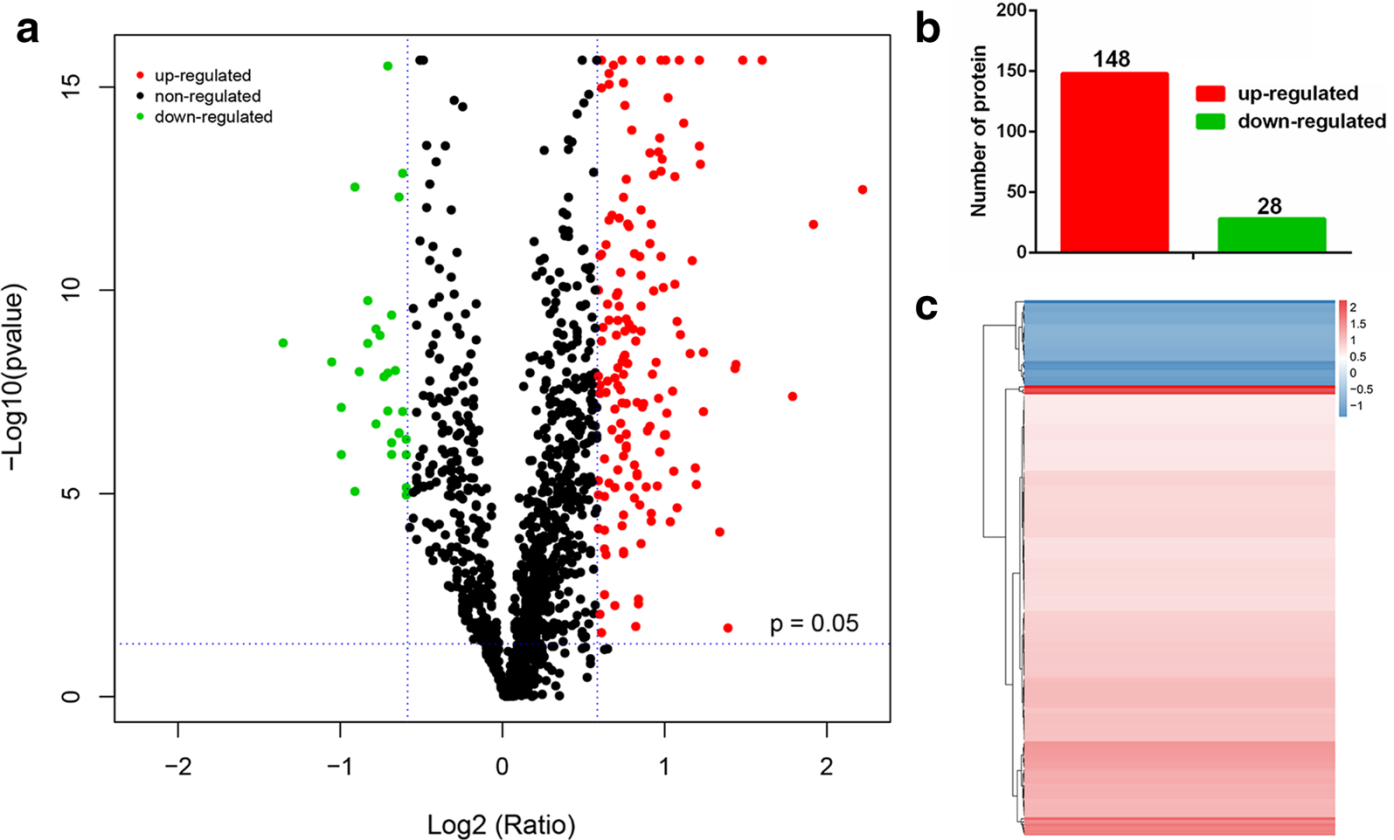
Venn and cluster results of IBT quantification between AIH and normal mice. **a** Volcano plot of differentially expressed proteins between the two groups (AIH vs. Normal). Each point represents a detected protein. The red dots indicate the up-regulated proteins, the green dots indicate the down-regulated proteins, and the gray dots indicate the non-significant differentially expressed proteins. This plot is a volcano plot of log2 fold-change (x-axis) versus − log10 Q-value (y-axis, representing the probability that the protein is differentially expressed). For the differentially expressed protein in a single test, a Q-value of < 0.05 and fold change of > 1.5 are set as the significant thresholds for differential expression. When the experiment was repeated, differentially expressed proteins were defined based on a 1.5-fold change (mean value of all comparison groups) and P-value (t-test of all comparison groups) of less than 0.05. **b** The number of significantly up-regulated proteins (148) and down-regulated proteins (28) in the AIH and normal groups are shown in the histogram. **c** Hierarchical cluster analysis results of DEPs between the AIH group and normal group. The blue color represents the down-regulated DEPs, the red color represents the up-regulated DEPs, and the white color represents the DEPs with no detectable expression change. The cluster analysis was conducted based on Euclidean distance and a hierarchical algorithm

To gain a more comprehensive understanding of AIH and determine the proteomic changes among AIH mice and normal mice, 5 AIH mice and 5 normal mice were sacrificed on day 35 and their plasma were collected for analysis using IBT quantification technology. Peptides of 6545 peptides and 1365 proteins were found in this experiment. Moreover, 176 DEPs were detected, and a volcano plot of these DEPs was generated (Fig. 5a). There were 148 up-regulated DEPs (red dots) and 28 down-regulated DEPs (green dots), while the expression of other proteins identified showed no difference (gray dots). The histogram of the up-regulated- and downregulated DEPs is shown in Fig. 5b. The hierarchical cluster result of the significantly regulated proteins is displayed in Fig. 5c. The right panel with color gradient represents the changes of protein abundances from down-regulated to up-regulated.

### GO enrichment analysis for DEPs

**Fig. 6:**
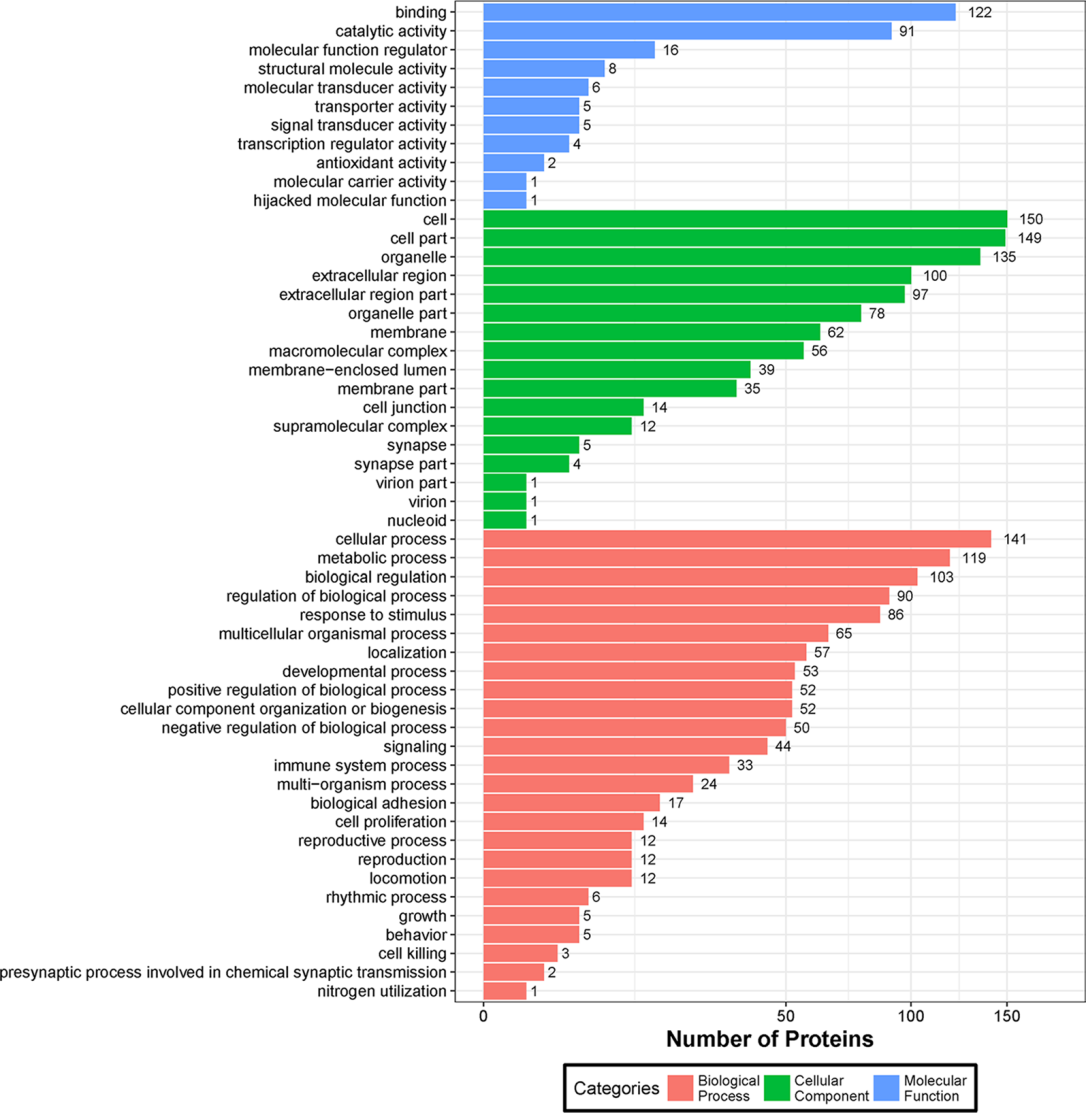
GO enrichment analysis of DEPs. A bar chart of the distribution of corresponding GO terms including three ontologies (cellular component, molecular function, and biological process) is presented. The numbers on the bar chart represent the corresponding number of proteins. An analysis was implemented based on all the identified DEPs in this part. DEPs were considered to be significantly regulated if the P-value was less than 0.05. In the GO enrichment analysis, hypergeometric test was used to obtain the target GO terms. GO analysis was performed using the software Blast2GO, and all identified proteins were compared to the corresponding NR database to obtain the GO function

The GO analysis covers three domains: (1) Biological process, series of events accomplished by one or more ordered assemblies of molecular functions, (2) Cell component, each component of the cells and the extracellular environment, and (3) Molecular function, activities, such as catalytic or binding activities, that occur at the molecular level. A bar plot depicting the analysis of these three ontologies is presented in Fig. 6. Fewer DEPs participated in molecular function than in cell component and biological processes. However, the molecular function analysis indicated that most DEPs were involved in the binding process and catalytic activity, which might play very important roles in immune cell interaction and antigen presentation. The cell, the cell part, the organelle, the extracellular region, and the extracellular region part are the top five cellular components, which may also be involved in several kinds of immune response. With regards to the biological process, the cellular process, single-organism process, metabolic process, and biological regulation involved the most DEPs. All significant terms (P < 0.01) of the three ontologies of DEPs are listed in the Additional file 4: Tables S2–S4.

### Pathway enrichment analysis of DEPs

**Fig. 7:**
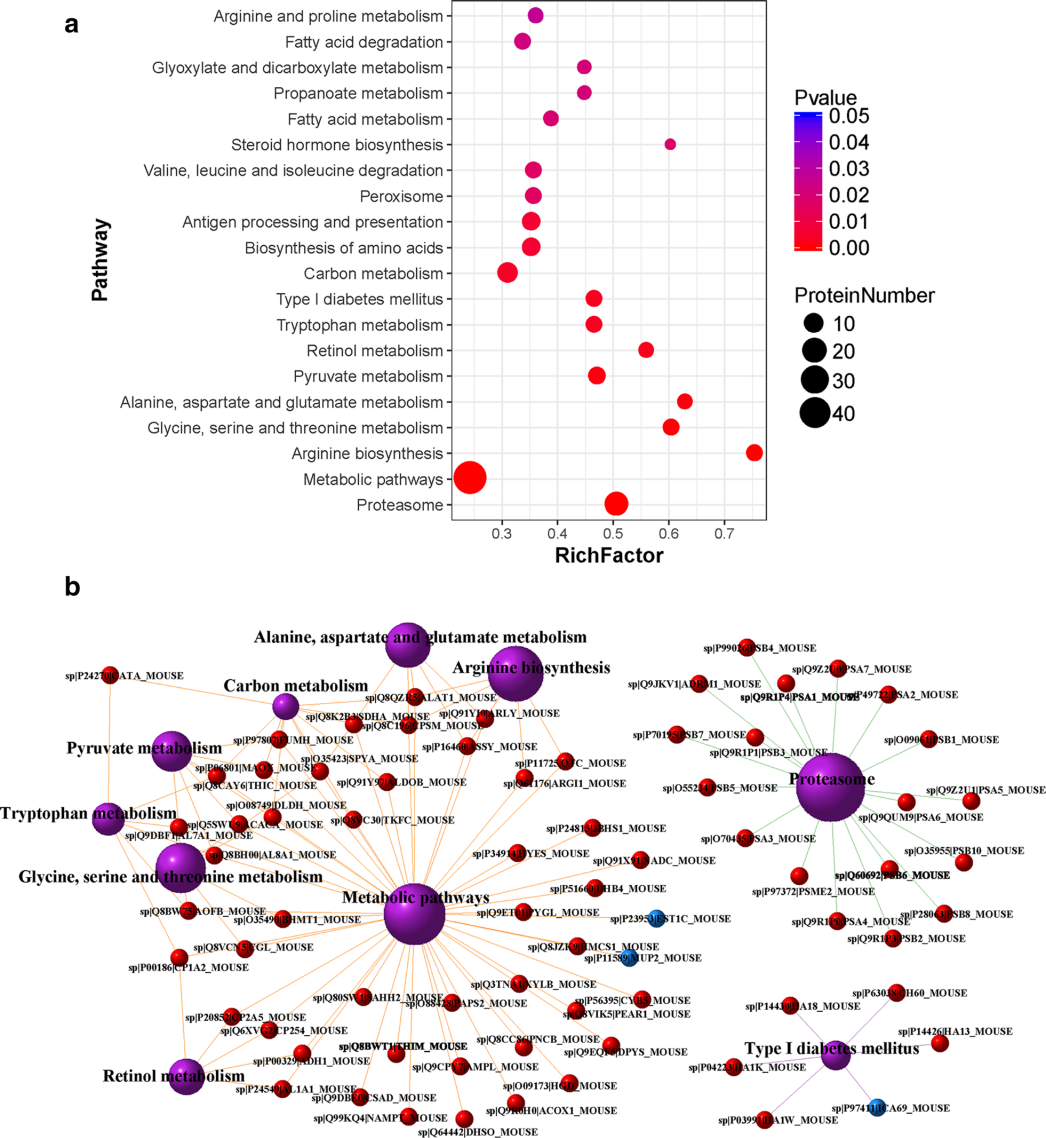
Pathway enrichment analysis of DEPs. **a** The screenshot of pathway enrichment analysis is shown. The top 20 enriched pathway terms are displayed. The P-value indicates the enrichment level of the pathway term and ranges from 0 to 1. A lesser P-value indicates greater intensiveness. Rich Factor is the ratio of the number of differentially expressed proteins annotated in the pathway term to all protein number annotated in this pathway term. A greater Rich Factor indicates greater intensiveness. **b** The network analysis of pathway terms. The ten purple balls represent the top 10 significant KEGG pathways. The red and blue balls represent the up- and down-regulated DEPs, respectively. The bigger ball represents the higher enrichment level of the relevant pathway. The edges with different colors represent the classes in the KEGG pathway analysis (sp number represents the swiss prot ID of the proteins; the gene name and species also showed on the ball; orange: metabolism; green: genetic information processing; purple: human disease)

Pathway enrichment analysis of DEPs based on the KEGG database was performed to analyze the interaction between proteins in certain biological functions. The DEPs were involved in 194 KEGG pathways and there were 30 significant KEGG pathways (P < 0.05) (Additional file 5: Table S5). The top 20 pathways and the relevant number of DEPs are listed in Fig. 7a. The bar plots of the pathway analysis for all DEPs are shown in Additional file 6: Figure S3. According to the data, metabolic pathways played the most important role in the process category and they involved the most DEPs. Moreover, the antigen processing and presentation pathways were detected as highly significant. Considering that antigen processing and presentation is important in immune response, especially in AIH, we displayed the map of this pathway in Additional file 7: Figure S4. In the analysis map, we found that the up-regulated DEPs, Heat shock protein (HSP) 70 and HSP90, participated in the major histocompatibility complex (MHC) I pathway, which could help enhance the function of CD8^+^T cells and NK cells. To gain more understanding of the interaction of different proteins in these pathways, a network analysis of the top 10 significant KEGG pathway terms and the relevant DEPs is presented in Fig. 7b. The DEPs marked in the network act as mediators between different pathways. For instance, HSP90 not only participated in antigen processing and presentation, but was also involved in many other pathways, such as protein processing in necroptosis, the IL-17 signaling pathway and Th17 cell differentiation pathways in cancer (data not shown), suggesting that it may be a key factor in AIH pathogenesis.

### Protein–protein interaction among vital DEPs

**Fig. 8:**
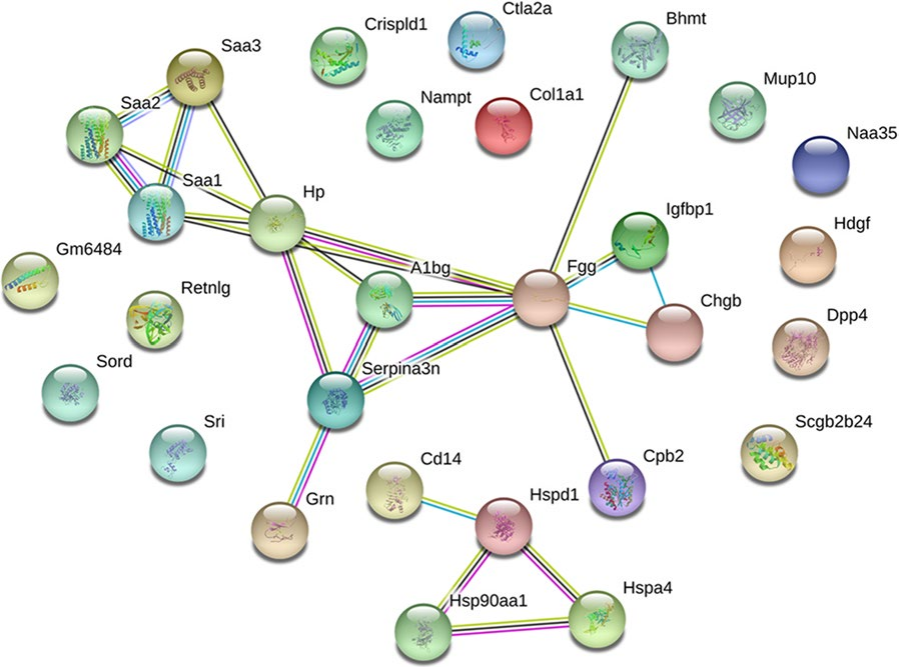
Protein–protein interaction of vital DEPs. The interactions between the several vital DEPs are displayed in the map. Every node represents a protein with its gene name marked. The structure in the filled nodes represents the known or the predicted 3D structure of the proteins. Edges with different colors represent protein–protein associations. The blue edges indicate the known interactions from curated databases, while the purple edges indicate the interaction between the proteins that have already been experimentally determined. Predicted interactions are indicated by green edges (gene neighborhood), red edges (gene fusions), and mazarine blue edges (gene co-occurrence). The other edges represent the extra nodes (yellow: text mining; black: co-expression; light blue: protein homology)

Proteins usually work as complexes by interacting with other molecules. To detect new biomarkers and identify the potential interaction between them, we first focused on several vital secretory DEPs. The fold changes of these DEPs and the corresponding gene names are listed in Additional file 8: Table S6. We analyzed the known and predicted PPIs of these DEPs using the STRING database (http://www.string-db.org) [26]. We then drew a map of the PPIs (Fig. 8). These proteins may work with each other through known or predicted interactions. For example, SAA1, SAA2, and SAA3 showed homology to one another and may be co-expressed with HP. According to the curated databases, SAA3 may work in combination with SAA1 or SAA2. A1BG seems to be the central, intermediary link for the listed DEPs. In addition, members of the HSP family such as HSP 60 and HSP90, together with CD14 formed another interaction map. Based on our results, we concluded that these vital proteins may play crucial roles in AIH pathogenesis and are likely to affect or work with one another.

### Validation of DEPs in the plasma of patients with AIH

**Fig. 9:**
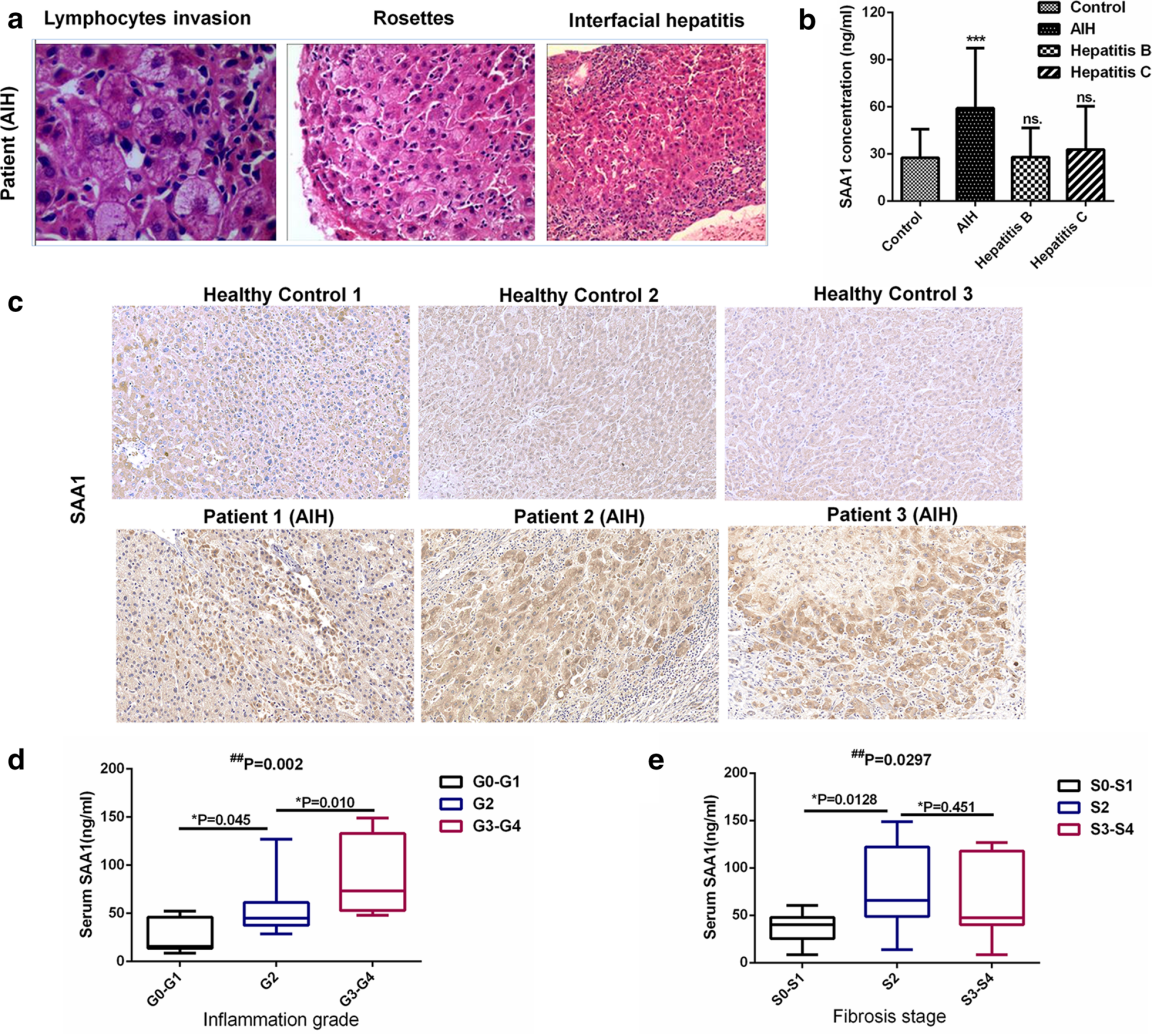
Vital DEPs validation in patients with AIH. **a** Representative H&E staining of liver tissue section from a patient with AIH. **b** Plasma from patients with AIH, hepatitis B patients, hepatitis C patients, and healthy people were collected and the SAA1 expression levels in the plasma were detected by ELISA (n = 30, ***P < 0.001 vs. Control). **c** Immunohistochemical staining of human liver tissues for SAA1 detection are shown (n = 3, ×200 magnification). **d** Box plot of plasma SAA1 level in patients with AIH from different inflammation grades (n = 7 for G0–G1, n = 14 for G2, n = 8 for G3–G4, ^#^Kruskal–Wallis nonparametric test *Mann–Whitney test). The line through the middle of each box represents the median. The length of the box represents the level range. **e** Box plot of plasma SAA1 level in patients with AIH from different fibrosis stages (n = 14 for S0–S1, n = 8 for S2, n = 7 for S3–S4, ^#^Kruskal–Wallis nonparametric test *Mann–Whitney test)

To further verify whether the DEPs detected in AIH mice changed in human patients, we collected plasma from 30 patients with AIH, 30 patients with hepatitis B, 30 patients with hepatitis C, and 30 healthy people (control). Patients with AIH were diagnosed using the IAIHG score and all the patients had liver biopsy results. Typical pathological features such as lymphocytes invasion, rosettes, and interfacial hepatitis observed in patients with AIH are displayed in Fig. 9a. According to the ELISA results, there was a significant difference in the expression of plasma SAA1 between patients with AIH and healthy subjects (Fig. 9b). The plasma level of SAA1 in patients with AIH was generally higher than that in healthy subjects. However, the plasma level of SAA1 did not increase in patients suffering from hepatitis B and hepatitis C. We also performed immunochemical analysis to detect SAA1 expression in the liver tissues of patients with AIH (Fig. 9c). SAA1 was expressed in the cytoplasm of hepatocytes and was secreted into the extracellular space. We next categorized the patients with AIH into different groups according to their liver biopsy results. For liver inflammation stage, patients were divided into three groups: absent or portal inflammation only (G0–G1), mild interface hepatitis (G2), and moderate or severe interface hepatitis (G3–G4). For liver fibrosis grade, patients were also divided into three groups: no or mild fibrosis (S0–S1), moderate fibrosis (S2), and severe fibrosis or cirrhosis (S3–S4). Moreover, the plasma expression of SAA1 in the patients with AIH of each group was detected. We found a significant difference between the various grades of inflammation, when comparing the plasma levels of SAA1 (Fig. 9d). Overall, the plasma level of SAA1 in patients suffering from AIH with moderate or severe interface hepatitis (G3–G4) was higher than that in the patients with absent or portal inflammation only (G0–G1). However, although plasma SAA1 level increased in patients with moderate fibrosis compared to those with no or mild fibrosis, it did not differ significantly between the S2 and S3–S4 groups (Fig. 9e), indicating that they cannot accurately predict the level of fibrosis in patients with AIH.

## Discussion

AIH is an organ-specific disease that is characterized by an autoimmune attack against hepatocytes [27]. The exact pathogenesis and relevant mechanisms of AIH remain largely unclear. Corticosteroids with or without azathioprine are the standard clinical therapy for patients with AIH. AIH shows a good response to immunosuppressant in most cases; however, it is associated with poor prognosis if the treatment is delayed [28]. Therefore, early intervention and accurate diagnosis of AIH are crucial. However, the severity of liver inflammation and fibrosis largely depends on liver biopsy. Recently, research on identifying biomarkers for AIH has increased, but there are a limited number of studies on this topic because of the difficulty in diagnosing AIH and lack of a steady chronic AIH animal model [29]. In 1992, Tiegs et al. established a T-cell-mediated acute hepatitis mouse model using ConA. However, in this model, there was no chronic inflammation, progressive fibrosis, and autoantibody production. Therefore, to gain a more comprehensive understanding of the pathogenesis of AIH, an appropriate mouse model is required.

In 2008, Christen et al. for the first time, established an AIH mouse model by disrupting immunotolerance in the mouse liver using adenovirus expressing human *CYP2D6* [13], which has been identified as the hepatocellular surface antigen recognized by anti-LKM-1 antibodies. However, several years later, the results from Matthias et al. somehow differed from those of Christen et al., who did not observe AIH in mice following the induction of adenovirus infection [10]. Although adenoviruses have high transduction efficiency, their transgene expression is transient. Moreover, adenoviruses can induce a strong immunogenic response; thus, multiple adenoviruses injections may interfere with the pathogenesis of AIH. Considering these factors, we injected a negative adenovirus vector once into the mouse to induce an acute inflammatory response, which can make the mouse sensitive to the autoantigen. Next, we transfected *CYP2D6* gene into mice hepatocytes several times using p*CYP2D6* plasmid via injections into the tail vein of mice, which is based on hydrodynamic technique [22, 30]. Persistent liver inflammation, observed as cellular infiltration, hepatic fibrosis, and necrosis was observed in our mouse model. More importantly, the fluorescent images indicated that the autoantibodies in our AIH mouse model were similar to those patients with type-2 AIH (Fig. 4a). These evidences proved that our AIH mouse model could mimic the pathogenesis and clinical characteristics of AIH in the human body; thus, it may be a useful tool for AIH research.

Early diagnosis and treatment of AIH could lead to a good prognosis of patients. The detection of autoantibodies such as anti-LKM-1, anti-ANA, and anti-SMA, is an important component of AIH diagnostic criteria developed by IAIHG [15]. However, these autoantibodies are not specific for AIH and can also be detected in many other autoimmune liver diseases, such as primary biliary cirrhosis, primary sclerosing cholangitis [31], and viral hepatitis [32]. Therefore, identifying new biomarkers that are specific to the diagnosis of AIH is crucial. Over the past decades, several proteomics-based analyses have been reported to identify the potential serological markers for AIH [18, 19, 29]. Ballot et al. first identified heterogeneous nuclear ribonucleoprotein A2/B1 as an autoantigen for type 1 AIH through proteomic analysis [33]. Phosphoglycerate mutase isozyme B (PGAM-B) was also identified as a biomarker for AIH through serum proteomic-based analysis in another study [19]. Moreover, Hongbin et al. conducted serum proteomic analysis of AIH in a ConA-induced hepatitis mouse model and found that the levels of the third component of complement (C3) and alpha-2-macroglobulin (A2M) increased both in mice with ConA-induced hepatitis and patients with AIH [29]. However, because of the heterogeneity of patients and the limitation in the number of blood samples collected from the patients for analysis, very few studies were conducted.

In the present study, we collected the plasma from our AIH mice models to detect the DEPs using IBT technology and then confirmed these findings in patients with AIH. The pathway and GO analyses were also conducted to gain some new insights into the pathogenesis of AIH. Via the GO analysis, we found that most DEPs were involved in the binding, cellular, and metabolic processes, which might play important roles in immune processes and in AIH pathogenesis. The most significant metabolic pathway based on DEPs was the arginine biosynthesis pathway. Arginine is crucial in host defense when invading pathogens are encountered; it can affect the balance of the Th1/Th2 response [34]. Studies have suggested that increased metabolism of l-arginine by myeloid cells can result in the impairment of lymphocyte responses [35, 36]. Moreover, in particular, the modifications of arginine within the context of a defined protein can lead to a specific B-cell immune response, that is closely related to the generation of antibodies [37]. These data suggest that several metabolic processes may also play a role in the pathogenesis of AIH, an aspect that has been rarely studied before. However, the most significant pathway was the proteasome pathway, which involved the second highest number of DEPs (Additional file 5: Table S5). The proteasome pathway is involved in many essential cellular functions, such as protein QC, immune responses, cell signaling, and apoptosis [38]. Increasing evidence shows that proteasome inhibitors interfere with antigen processing and presentation, and antibody production. Moreover, they block the signaling cascades in immune cell function and survival [39]. In clinical practice, proteasome inhibitors have been used in the treatment of several autoimmune diseases [40]. Therefore, we assume from these results that proteasome inhibitors could also be a novel strategy for AIH treatment in the future.

Based on the analysis of the DEPs, we found that the members of the serum amyloid A protein family (SAA1, SAA2, and SAA3) were the most abundant DEPs (Additional file 8: Table S6). The SAA proteins represent a family of proteins that are acute phase respondents [41]. SAA1 is the most widely expressed, the best-characterized, and, the most active SAA protein, which is a component of, and is secreted by, hepatocytes [20]. Recently, SAA1 has been found to play key roles in bacterial clearance, immune regulation, and tumor pathogenesis [42, 43], and it was also identified as an important link between mucosal T cells, microbial communities, and their tissue environments in patients with inflammatory bowel disease [44]. An analysis based on iTRAQ revealed that SAA1 was up-regulated in HBV-related HCC [45]. Moreover, SAA1 and HP, which were found to be upregulated DEPs in our results, play important roles in the surveillance of HCC in patients with an early stage of cirrhosis [46]. Young et al. further used SAA1 transgenic mouse model and found that SAA1 aggravates ConA-induced T cell-mediated hepatitis. However, the role of SAA1 in chronic hepatitis has not yet been clearly elucidated. Our results revealed that the expression of SAA1 in AIH mice was three times higher than that in normal mice. We also verified this finding in the plasma and the liver tissues of patients with AIH, suggesting that SAA1 may indeed be involved in immune disorders associated with AIH. Moreover, compared to patients suffering from hepatitis B and hepatitis C, SAA1 increased more specifically in patients with AIH (Fig. 9c) and a higher level of SAA1 was related with more severe inflammation based on the modified Scheuer histologic scoring system. SAA1 might also increase in patients with AIH with severe fibrosis but it might not accurately predict the state of fibrosis. Combining our results with the above description, we speculate that SAA1 may serve as a potential plasma biomarker for patients with AIH. However, considering the complex etiology and mechanism of AIH, further studies should be performed to reinforce the findings of the present study.

## Conclusions

In summary, these proteomic results suggest some novel ideas for AIH diagnosis, pathogenesis, and treatment based on our improved AIH mouse model. In addition, we further confirmed that SAA1, the most significant DEPs in our study, was increased in the plasma of patients with AIH and might be a biomarker for the clinical diagnosis of AIH.

## Supplementary information

**Additional file 1: Figure S1.** A diagram for the establishment of the AIH mouse model. The upward arrows mean the time for injection through tail vein and the downward arrows mean the time for mice sacrifice.

**Additional file 2: Table S1.** Primer Sequences for polymerase chain reaction.

**Additional file 3: Figure S2.** Representative H&E stain pictures of control groups for the mouse model (200× and 400× magnification)

**Additional file 4: Table S2–S4.** The highly significantly enrichment GO terms (with the P value < 0.01) of the cell component (Table S2), molecular function (Table S3), and biological process (Table S4).

**Additional file 5: Table S5.** The significant KEGG pathways (P-value < 0.05) involved by DEPs.

**Additional file 6: Figure S3.***A*) A barplot of pathway analysis for all DEPs. *B*) The up- and down-regulated DEPs in the significant pathways. Red means up-regulated proteins and blue means down-regulated proteins; X-axis displays the name of pathway; Y-axis displays the count of differentially expressed protein.

**Additional file 7: Figure S4.** The pathway map of antigen processing and presentation. Red means up-regulated and green means down-regulated DEPs.

**Additional file 8: Table S6.** Description and the mean ratio of the DEPs listed in Fig. 8. The comparison was conducted between the AIH mice group and normal mice group. Mean Ratio, SD, and P-value represent the statistical data of AIH mice vs. Normal mice by IBT (n = 5).

## Data Availability

The main data generated or analyzed during this study are included in this manuscript and its Additional files. Other proteomic data mentioned in this manuscript are available from the corresponding author on reasonable request.

## Abbreviations

AIH: autoimmune hepatitis
PBC: primary biliary cirrhosis
IAIHG: International Autoimmune Hepatitis Group
CYP2D6: cytochrome P450 2D6
Ad-2D6: adenovirus expressing human cytochrome P450 2D6
pCYP2D6: cytochrome P450 2D6 plasmid
ConA: concanavalin A
ANA: antinuclear antibody
AMA: anti-mitochondrial antibody
LKM-1: anti-liver-kidney microsomes antibody
MHC: major histocompatibility complex
IBT: isobaric tags
iTRAQ: isobaric tags for relative and absolute quantitation
GO: gene ontology
KEGG: kyoto encyclopedia of genes and genomes
DEP: different expressed proteins
SAA: serum amyloid A 1
HSP: heat shock protein
A2M: alpha-2-macroglobulin
C3: component of complement
