# Supplementary material for: Plasma proteomic analysis of autoimmune hepatitis in an improved AIH mouse model
Source: J Transl Med. 2020 Jan 6;18:3. doi: 10.1186/s12967-019-02180-3 (PMC6943959; doi:10.1186/s12967-019-02180-3)
Supplement: Supplementary file 1 — Additional file 1: Figure S1. A diagram for the establishment of the AIH mouse model. The upward arrows mean the time for injection through tail vein and the downward arrows mean the time for mice sacrifice. [file 12967_2019_2180_MOESM1_ESM.docx]

**Additional file 1: Figure S1** A diagram for the establishment of the AIH mouse model. The upward arrows mean the time for injection through tail vein and the downward arrows mean the time for sacrifice.

**
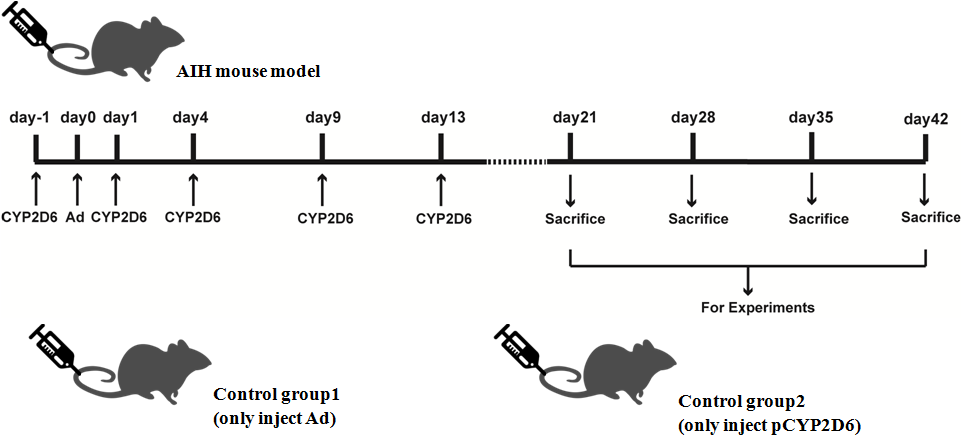
**
