# Supplementary material for: Plasma proteomic analysis of autoimmune hepatitis in an improved AIH mouse model
Source: J Transl Med. 2020 Jan 6;18:3. doi: 10.1186/s12967-019-02180-3 (PMC6943959; doi:10.1186/s12967-019-02180-3)
Supplement: Supplementary file 2 — Additional file 2: Table S1. Primer Sequences for polymerase chain reaction. [file 12967_2019_2180_MOESM2_ESM.docx]

**Additional file 2: Table S1:** Primer Sequences for polymerase chain reaction.

| Genes | Sequences |
| --- | --- |
| CYP2D6 | **F:5’-TGGCAAGGTCCTACGCTTC-3’**  **R:5’-GCCACCACTATGCACAGGTT-3’** |
| GAPDH | **F:5’-AGGTCGGTGTGAACGGATTTG-3’**  **R:5’-TGTAGACCATGTAGTTGAGGTCA -3’** |
