# Supplementary material for: Plasma proteomic analysis of autoimmune hepatitis in an improved AIH mouse model
Source: J Transl Med. 2020 Jan 6;18:3. doi: 10.1186/s12967-019-02180-3 (PMC6943959; doi:10.1186/s12967-019-02180-3)

**Additional file 3: Figure S2** Representative H&E stain pictures of control groups for the mouse model (200× and 400× magnification).


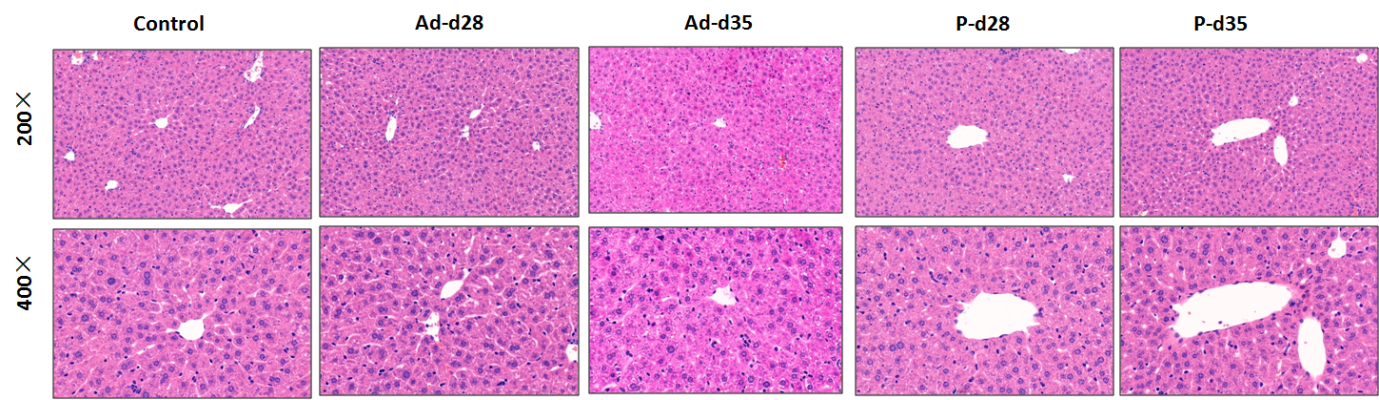

Supplement: Supplementary file 3 — Additional file 3: Figure S2. Representative H&E stain pictures of control groups for the mouse model (200× and 400× magnification) [file 12967_2019_2180_MOESM3_ESM.docx]
