# Supplementary material for: Plasma proteomic analysis of autoimmune hepatitis in an improved AIH mouse model
Source: J Transl Med. 2020 Jan 6;18:3. doi: 10.1186/s12967-019-02180-3 (PMC6943959; doi:10.1186/s12967-019-02180-3)
Supplement: Supplementary file 4 — Additional file 4: Table S2–S4. The highly significantly enrichment GO terms (with the P value < 0.01) of the cell component (Table S2), molecular function (Table S3), and biological process (Table S4). [file 12967_2019_2180_MOESM4_ESM.docx]

**Additional file 4: Table S2:** The highly significantly enrichment GO terms of the cell component (with the P value<0.01).

| **Gene Ontology term** | **Cluster frequency** | **Protein frequency of use** | **P-value** |
| --- | --- | --- | --- |
| [**proteasome core complex**](http://amigo.geneontology.org/amigo/term/GO:0005839) | 16 out of 159 genes, 10.1% | 18 out of 1227 genes, 1.5% | 3.86247e-13 |
| [**proteasome complex**](http://amigo.geneontology.org/amigo/term/GO:0000502) | 18 out of 159 genes, 11.3% | 47 out of 1227 genes, 3.8% | 6.650469e-06 |
| [**proteasome core complex, alpha-subunit complex**](http://amigo.geneontology.org/amigo/term/GO:0019773) | 7 out of 159 genes, 4.4% | 9 out of 1227 genes, 0.7% | 1.560499e-05 |
| [**mitochondrial part**](http://amigo.geneontology.org/amigo/term/GO:0044429) | 19 out of 159 genes, 11.9% | 60 out of 1227 genes, 4.9% | 8.478971e-05 |
| [**mitochondrial envelope**](http://amigo.geneontology.org/amigo/term/GO:0005740) | 16 out of 159 genes, 10.1% | 47 out of 1227 genes, 3.8% | 0.0001226314 |
| [**mitochondrial membrane**](http://amigo.geneontology.org/amigo/term/GO:0031966) | 15 out of 159 genes, 9.4% | 44 out of 1227 genes, 3.6% | 0.0001992829 |
| [**mitochondrion**](http://amigo.geneontology.org/amigo/term/GO:0005739) | 35 out of 159 genes, 22.0% | 174 out of 1227 genes, 14.2% | 0.00268471 |
| [**mitochondrial inner membrane**](http://amigo.geneontology.org/amigo/term/GO:0005743) | 10 out of 159 genes, 6.3% | 30 out of 1227 genes, 2.4% | 0.002974959 |
| [**organelle inner membrane**](http://amigo.geneontology.org/amigo/term/GO:0019866) | 10 out of 159 genes, 6.3% | 30 out of 1227 genes, 2.4% | 0.002974959 |
| [**organelle membrane**](http://amigo.geneontology.org/amigo/term/GO:0031090) | 32 out of 159 genes, 20.1% | 160 out of 1227 genes, 13.0% | 0.004658868 |
| [**membrane-bounded organelle**](http://amigo.geneontology.org/amigo/term/GO:0043227) | 106 out of 159 genes, 66.7% | 707 out of 1227 genes, 57.6% | 0.008004483 |
| [**intracellular membrane-bounded organelle**](http://amigo.geneontology.org/amigo/term/GO:0043231) | 106 out of 159 genes, 66.7% | 707 out of 1227 genes, 57.6% | 0.008004483 |
| [**microbody part**](http://amigo.geneontology.org/amigo/term/GO:0044438) | 5 out of 159 genes, 3.1% | 11 out of 1227 genes, 0.9% | 0.008248682 |
| [**peroxisomal part**](http://amigo.geneontology.org/amigo/term/GO:0044439) | 5 out of 159 genes, 3.1% | 11 out of 1227 genes, 0.9% | 0.008248682 |

**Additional file 4: Table S3:** The top significantly enrichment GO terms of molecular function (P-value<0.01).

| **Gene Ontology term** | **Cluster frequency** | **Protein frequency of use** | **P-value** |
| --- | --- | --- | --- |
| [**threonine-type endopeptidase activity**](http://amigo.geneontology.org/amigo/term/GO:0004298) | 16 out of 154 genes, 10.4% | 18 out of 1192 genes, 1.5% | 3.601377e-13 |
| [**threonine-type peptidase activity**](http://amigo.geneontology.org/amigo/term/GO:0070003) | 16 out of 154 genes, 10.4% | 18 out of 1192 genes, 1.5% | 3.601377e-13 |
| [**pyridoxal phosphate binding**](http://amigo.geneontology.org/amigo/term/GO:0030170) | 6 out of 154 genes, 3.9% | 11 out of 1192 genes, 0.9% | 0.001122204 |
| [**cofactor binding**](http://amigo.geneontology.org/amigo/term/GO:0048037) | 16 out of 154 genes, 10.4% | 57 out of 1192 genes, 4.8% | 0.001381808 |
| [**nicotinate-nucleotide diphosphorylase (carboxylating) activity**](http://amigo.geneontology.org/amigo/term/GO:0004514) | 3 out of 154 genes, 1.9% | 3 out of 1192 genes, 0.3% | 0.002119926 |
| [**toxic substance binding**](http://amigo.geneontology.org/amigo/term/GO:0015643) | 3 out of 154 genes, 1.9% | 3 out of 1192 genes, 0.3% | 0.002119926 |
| [**lipopolysaccharide binding**](http://amigo.geneontology.org/amigo/term/GO:0001530) | 4 out of 154 genes, 2.6% | 6 out of 1192 genes, 0.5% | 0.003265215 |
| [**heme binding**](http://amigo.geneontology.org/amigo/term/GO:0020037) | 7 out of 154 genes, 4.5% | 19 out of 1192 genes, 1.6% | 0.006832343 |
| [**tetrapyrrole binding**](http://amigo.geneontology.org/amigo/term/GO:0046906) | 7 out of 154 genes, 4.5% | 20 out of 1192 genes, 1.7% | 0.009390534 |
| [**endopeptidase activity**](http://amigo.geneontology.org/amigo/term/GO:0004175) | 19 out of 154 genes, 12.3% | 86 out of 1192 genes, 7.2% | 0.009907244 |

**Additional file 4: Table S4:** The top significant GO enrichment terms of biological process (P-value<0.01).

| **Gene Ontology term** | **Cluster frequency** | **Protein frequency of use** | **P-value** |
| --- | --- | --- | --- |
| [**small molecule catabolic process**](http://amigo.geneontology.org/amigo/term/GO:0044282) | 17 out of 152 genes, 11.2% | 44 out of 1204 genes, 3.7% | 7.307024e-06 |
| [**single-organism catabolic process**](http://amigo.geneontology.org/amigo/term/GO:0044712) | 17 out of 152 genes, 11.2% | 44 out of 1204 genes, 3.7% | 7.307024e-06 |
| [**antigen processing and presentation of exogenous peptide antigen via MHC class I**](http://amigo.geneontology.org/amigo/term/GO:0042590) | 13 out of 152 genes, 8.6% | 31 out of 1204 genes, 2.6% | 3.402306e-05 |
| [**antigen processing and presentation of peptide antigen via MHC class I**](http://amigo.geneontology.org/amigo/term/GO:0002474) | 13 out of 152 genes, 8.6% | 33 out of 1204 genes, 2.7% | 7.514899e-05 |
| [**antigen processing and presentation of exogenous peptide antigen**](http://amigo.geneontology.org/amigo/term/GO:0002478) | 13 out of 152 genes, 8.6% | 35 out of 1204 genes, 2.9% | 0.0001538568 |
| [**urea cycle**](http://amigo.geneontology.org/amigo/term/GO:0000050) | 5 out of 152 genes, 3.3% | 6 out of 1204 genes, 0.5% | 0.000162999 |
| [**urea metabolic process**](http://amigo.geneontology.org/amigo/term/GO:0019627) | 5 out of 152 genes, 3.3% | 6 out of 1204 genes, 0.5% | 0.000162999 |
| [**nitrogen cycle metabolic process**](http://amigo.geneontology.org/amigo/term/GO:0071941) | 5 out of 152 genes, 3.3% | 6 out of 1204 genes, 0.5% | 0.000162999 |
| [**protein catabolic process**](http://amigo.geneontology.org/amigo/term/GO:0030163) | 19 out of 152 genes, 12.5% | 65 out of 1204 genes, 5.4% | 0.0001950433 |
| [**cellular protein catabolic process**](http://amigo.geneontology.org/amigo/term/GO:0044257) | 18 out of 152 genes, 11.8% | 60 out of 1204 genes, 5.0% | 0.0002021747 |
| [**proteolysis involved in cellular protein catabolic process**](http://amigo.geneontology.org/amigo/term/GO:0051603) | 18 out of 152 genes, 11.8% | 60 out of 1204 genes, 5.0% | 0.0002021747 |
| [**antigen processing and presentation of exogenous antigen**](http://amigo.geneontology.org/amigo/term/GO:0019884) | 13 out of 152 genes, 8.6% | 36 out of 1204 genes, 3.0% | 0.0002147064 |
| [**antigen processing and presentation of peptide antigen**](http://amigo.geneontology.org/amigo/term/GO:0048002) | 13 out of 152 genes, 8.6% | 36 out of 1204 genes, 3.0% | 0.0002147064 |
| [**DNA damage response, signal transduction by p53 class mediator**](http://amigo.geneontology.org/amigo/term/GO:0030330) | 10 out of 152 genes, 6.6% | 24 out of 1204 genes, 2.0% | 0.0003130198 |
| [**mitotic G1 DNA damage checkpoint**](http://amigo.geneontology.org/amigo/term/GO:0031571) | 10 out of 152 genes, 6.6% | 24 out of 1204 genes, 2.0% | 0.0003130198 |
| [**signal transduction in response to DNA damage**](http://amigo.geneontology.org/amigo/term/GO:0042770) | 10 out of 152 genes, 6.6% | 24 out of 1204 genes, 2.0% | 0.0003130198 |
| [**signal transduction by p53 class mediator**](http://amigo.geneontology.org/amigo/term/GO:0072331) | 10 out of 152 genes, 6.6% | 24 out of 1204 genes, 2.0% | 0.0003130198 |
| [**positive regulation of cell cycle process**](http://amigo.geneontology.org/amigo/term/GO:0090068) | 11 out of 152 genes, 7.2% | 29 out of 1204 genes, 2.4% | 0.0004134737 |
| [**mitotic cell cycle checkpoint**](http://amigo.geneontology.org/amigo/term/GO:0007093) | 10 out of 152 genes, 6.6% | 25 out of 1204 genes, 2.1% | 0.0004656078 |
| [**mitotic DNA damage checkpoint**](http://amigo.geneontology.org/amigo/term/GO:0044773) | 10 out of 152 genes, 6.6% | 25 out of 1204 genes, 2.1% | 0.0004656078 |
| [**mitotic DNA integrity checkpoint**](http://amigo.geneontology.org/amigo/term/GO:0044774) | 10 out of 152 genes, 6.6% | 25 out of 1204 genes, 2.1% | 0.0004656078 |
| [**isoprenoid biosynthetic process**](http://amigo.geneontology.org/amigo/term/GO:0008299) | 5 out of 152 genes, 3.3% | 7 out of 1204 genes, 0.6% | 0.0005123586 |
| [**organic acid catabolic process**](http://amigo.geneontology.org/amigo/term/GO:0016054) | 12 out of 152 genes, 7.9% | 35 out of 1204 genes, 2.9% | 0.0006619511 |
| [**carboxylic acid catabolic process**](http://amigo.geneontology.org/amigo/term/GO:0046395) | 12 out of 152 genes, 7.9% | 35 out of 1204 genes, 2.9% | 0.0006619511 |
| [**regulation of cellular amine metabolic process**](http://amigo.geneontology.org/amigo/term/GO:0033238) | 10 out of 152 genes, 6.6% | 26 out of 1204 genes, 2.2% | 0.0006753112 |
| [**regulation of cellular amino acid metabolic process**](http://amigo.geneontology.org/amigo/term/GO:0006521) | 9 out of 152 genes, 5.9% | 22 out of 1204 genes, 1.8% | 0.0007496093 |
| [**carboxylic acid metabolic process**](http://amigo.geneontology.org/amigo/term/GO:0019752) | 30 out of 152 genes, 19.7% | 136 out of 1204 genes, 11.3% | 0.0007546906 |
| [**DNA damage checkpoint**](http://amigo.geneontology.org/amigo/term/GO:0000077) | 10 out of 152 genes, 6.6% | 27 out of 1204 genes, 2.2% | 0.0009573731 |
| [**DNA integrity checkpoint**](http://amigo.geneontology.org/amigo/term/GO:0031570) | 10 out of 152 genes, 6.6% | 27 out of 1204 genes, 2.2% | 0.0009573731 |
| [**cellular hormone metabolic process**](http://amigo.geneontology.org/amigo/term/GO:0034754) | 6 out of 152 genes, 3.9% | 11 out of 1204 genes, 0.9% | 0.0009892429 |
| [**response to cadmium ion**](http://amigo.geneontology.org/amigo/term/GO:0046686) | 6 out of 152 genes, 3.9% | 11 out of 1204 genes, 0.9% | 0.0009892429 |
