# Supplementary material for: Plasma proteomic analysis of autoimmune hepatitis in an improved AIH mouse model
Source: J Transl Med. 2020 Jan 6;18:3. doi: 10.1186/s12967-019-02180-3 (PMC6943959; doi:10.1186/s12967-019-02180-3)
Supplement: Supplementary file 5 — Additional file 5: Table S5. The significant KEGG pathways (P-value < 0.05) involved by DEPs. [file 12967_2019_2180_MOESM5_ESM.docx]

**Additional file 5: Table S5** The significant KEGG pathways (P-value<0.05) involved by DEPs.

| **AIH-VS-Normal** | | | | | |
| --- | --- | --- | --- | --- | --- |
| **#** | **Pathway** | **Diff Proteins with pathway annotation (171)** | **All Proteins with pathway annotation (1295)** | **P-value** | **Pathway ID** |
| **1** | [**Proteasome**](file:///E:\001%20doge\%E6%B5%8B%E5%BA%8F-%E5%8D%8E%E5%A4%A7\%E7%BB%93%E6%9E%9C\report\BGI_result\Function_Analyse\Enrichment_Analyse\Pathway_Enrichment\AIH-VS-Normal_Pathway_enrichment\AIH-VS-Normal.htm#gene1) | 17 (9.94%) | 35 (2.7%) | 2.823524e-07 | ko03050 |
| **2** | [**Metabolic pathways**](file:///E:\001%20doge\%E6%B5%8B%E5%BA%8F-%E5%8D%8E%E5%A4%A7\%E7%BB%93%E6%9E%9C\report\BGI_result\Function_Analyse\Enrichment_Analyse\Pathway_Enrichment\AIH-VS-Normal_Pathway_enrichment\AIH-VS-Normal.htm#gene2) | 47 (27.49%) | 204 (15.75%) | 1.674333e-05 | ko01100 |
| **3** | [**Arginine biosynthesis**](file:///E:\001%20doge\%E6%B5%8B%E5%BA%8F-%E5%8D%8E%E5%A4%A7\%E7%BB%93%E6%9E%9C\report\BGI_result\Function_Analyse\Enrichment_Analyse\Pathway_Enrichment\AIH-VS-Normal_Pathway_enrichment\AIH-VS-Normal.htm#gene3) | 6 (3.51%) | 8 (0.62%) | 0.0001089427 | ko00220 |
| **4** | [**Alanine, aspartate and glutamate metabolism**](file:///E:\001%20doge\%E6%B5%8B%E5%BA%8F-%E5%8D%8E%E5%A4%A7\%E7%BB%93%E6%9E%9C\report\BGI_result\Function_Analyse\Enrichment_Analyse\Pathway_Enrichment\AIH-VS-Normal_Pathway_enrichment\AIH-VS-Normal.htm#gene4) | 6 (3.51%) | 9 (0.69%) | 0.0002911073 | ko00250 |
| **5** | [**Glycine, serine and threonine metabolism**](file:///E:\001%20doge\%E6%B5%8B%E5%BA%8F-%E5%8D%8E%E5%A4%A7\%E7%BB%93%E6%9E%9C\report\BGI_result\Function_Analyse\Enrichment_Analyse\Pathway_Enrichment\AIH-VS-Normal_Pathway_enrichment\AIH-VS-Normal.htm#gene5) | 6 (3.51%) | 10 (0.77%) | 0.0006483895 | ko00260 |
| **6** | [**Pyruvate metabolism**](file:///E:\001%20doge\%E6%B5%8B%E5%BA%8F-%E5%8D%8E%E5%A4%A7\%E7%BB%93%E6%9E%9C\report\BGI_result\Function_Analyse\Enrichment_Analyse\Pathway_Enrichment\AIH-VS-Normal_Pathway_enrichment\AIH-VS-Normal.htm#gene6) | 7 (4.09%) | 15 (1.16%) | 0.001579125 | ko00620 |
| **7** | [**Biosynthesis of amino acids**](file:///E:\001%20doge\%E6%B5%8B%E5%BA%8F-%E5%8D%8E%E5%A4%A7\%E7%BB%93%E6%9E%9C\report\BGI_result\Function_Analyse\Enrichment_Analyse\Pathway_Enrichment\AIH-VS-Normal_Pathway_enrichment\AIH-VS-Normal.htm#gene7) | 9 (5.26%) | 24 (1.85%) | 0.002200067 | ko01230 |
| **8** | [**Retinol metabolism**](file:///E:\001%20doge\%E6%B5%8B%E5%BA%8F-%E5%8D%8E%E5%A4%A7\%E7%BB%93%E6%9E%9C\report\BGI_result\Function_Analyse\Enrichment_Analyse\Pathway_Enrichment\AIH-VS-Normal_Pathway_enrichment\AIH-VS-Normal.htm#gene8) | 5 (2.92%) | 9 (0.69%) | 0.003058968 | ko00830 |
| **9** | [**Tryptophan metabolism**](file:///E:\001%20doge\%E6%B5%8B%E5%BA%8F-%E5%8D%8E%E5%A4%A7\%E7%BB%93%E6%9E%9C\report\BGI_result\Function_Analyse\Enrichment_Analyse\Pathway_Enrichment\AIH-VS-Normal_Pathway_enrichment\AIH-VS-Normal.htm#gene9) | 6 (3.51%) | 13 (1%) | 0.003752778 | ko00380 |
| **10** | [**Type I diabetes mellitus**](file:///E:\001%20doge\%E6%B5%8B%E5%BA%8F-%E5%8D%8E%E5%A4%A7\%E7%BB%93%E6%9E%9C\report\BGI_result\Function_Analyse\Enrichment_Analyse\Pathway_Enrichment\AIH-VS-Normal_Pathway_enrichment\AIH-VS-Normal.htm#gene10) | 6 (3.51%) | 13 (1%) | 0.003752778 | ko04940 |
| **11** | [**Carbon metabolism**](file:///E:\001%20doge\%E6%B5%8B%E5%BA%8F-%E5%8D%8E%E5%A4%A7\%E7%BB%93%E6%9E%9C\report\BGI_result\Function_Analyse\Enrichment_Analyse\Pathway_Enrichment\AIH-VS-Normal_Pathway_enrichment\AIH-VS-Normal.htm#gene11) | 11 (6.43%) | 36 (2.78%) | 0.004693343 | ko01200 |
| **12** | [**Fatty acid metabolism**](file:///E:\001%20doge\%E6%B5%8B%E5%BA%8F-%E5%8D%8E%E5%A4%A7\%E7%BB%93%E6%9E%9C\report\BGI_result\Function_Analyse\Enrichment_Analyse\Pathway_Enrichment\AIH-VS-Normal_Pathway_enrichment\AIH-VS-Normal.htm#gene12) | 6 (3.51%) | 14 (1.08%) | 0.005857427 | ko01212 |
| **13** | [**Antigen processing and presentation**](file:///E:\001%20doge\%E6%B5%8B%E5%BA%8F-%E5%8D%8E%E5%A4%A7\%E7%BB%93%E6%9E%9C\report\BGI_result\Function_Analyse\Enrichment_Analyse\Pathway_Enrichment\AIH-VS-Normal_Pathway_enrichment\AIH-VS-Normal.htm#gene13) | 8 (4.68%) | 23 (1.78%) | 0.006632057 | ko04612 |
| **14** | [**Peroxisome**](file:///E:\001%20doge\%E6%B5%8B%E5%BA%8F-%E5%8D%8E%E5%A4%A7\%E7%BB%93%E6%9E%9C\report\BGI_result\Function_Analyse\Enrichment_Analyse\Pathway_Enrichment\AIH-VS-Normal_Pathway_enrichment\AIH-VS-Normal.htm#gene14) | 6 (3.51%) | 17 (1.31%) | 0.01715791 | ko04146 |
| **15** | [**Valine, leucine and isoleucine degradation**](file:///E:\001%20doge\%E6%B5%8B%E5%BA%8F-%E5%8D%8E%E5%A4%A7\%E7%BB%93%E6%9E%9C\report\BGI_result\Function_Analyse\Enrichment_Analyse\Pathway_Enrichment\AIH-VS-Normal_Pathway_enrichment\AIH-VS-Normal.htm#gene15) | 6 (3.51%) | 17 (1.31%) | 0.01715791 | ko00280 |
| **16** | [**Steroid hormone biosynthesis**](file:///E:\001%20doge\%E6%B5%8B%E5%BA%8F-%E5%8D%8E%E5%A4%A7\%E7%BB%93%E6%9E%9C\report\BGI_result\Function_Analyse\Enrichment_Analyse\Pathway_Enrichment\AIH-VS-Normal_Pathway_enrichment\AIH-VS-Normal.htm#gene16) | 3 (1.75%) | 5 (0.39%) | 0.01848039 | ko00140 |
| **17** | [**Propanoate metabolism**](file:///E:\001%20doge\%E6%B5%8B%E5%BA%8F-%E5%8D%8E%E5%A4%A7\%E7%BB%93%E6%9E%9C\report\BGI_result\Function_Analyse\Enrichment_Analyse\Pathway_Enrichment\AIH-VS-Normal_Pathway_enrichment\AIH-VS-Normal.htm#gene17) | 4 (2.34%) | 9 (0.69%) | 0.02162187 | ko00640 |
| **18** | [**Glyoxylate and dicarboxylate metabolism**](file:///E:\001%20doge\%E6%B5%8B%E5%BA%8F-%E5%8D%8E%E5%A4%A7\%E7%BB%93%E6%9E%9C\report\BGI_result\Function_Analyse\Enrichment_Analyse\Pathway_Enrichment\AIH-VS-Normal_Pathway_enrichment\AIH-VS-Normal.htm#gene18) | 4 (2.34%) | 9 (0.69%) | 0.02162187 | ko00630 |
| **19** | [**Fatty acid degradation**](file:///E:\001%20doge\%E6%B5%8B%E5%BA%8F-%E5%8D%8E%E5%A4%A7\%E7%BB%93%E6%9E%9C\report\BGI_result\Function_Analyse\Enrichment_Analyse\Pathway_Enrichment\AIH-VS-Normal_Pathway_enrichment\AIH-VS-Normal.htm#gene19) | 6 (3.51%) | 18 (1.39%) | 0.0229831 | ko00071 |
| **20** | [**Arginine and proline metabolism**](file:///E:\001%20doge\%E6%B5%8B%E5%BA%8F-%E5%8D%8E%E5%A4%A7\%E7%BB%93%E6%9E%9C\report\BGI_result\Function_Analyse\Enrichment_Analyse\Pathway_Enrichment\AIH-VS-Normal_Pathway_enrichment\AIH-VS-Normal.htm#gene20) | 5 (2.92%) | 14 (1.08%) | 0.02786749 | ko00330 |
| **21** | [**Allograft rejection**](file:///E:\001%20doge\%E6%B5%8B%E5%BA%8F-%E5%8D%8E%E5%A4%A7\%E7%BB%93%E6%9E%9C\report\BGI_result\Function_Analyse\Enrichment_Analyse\Pathway_Enrichment\AIH-VS-Normal_Pathway_enrichment\AIH-VS-Normal.htm#gene21) | 10 (5.85%) | 40 (3.09%) | 0.02975098 | ko05330 |
| **22** | [**Graft-versus-host disease**](file:///E:\001%20doge\%E6%B5%8B%E5%BA%8F-%E5%8D%8E%E5%A4%A7\%E7%BB%93%E6%9E%9C\report\BGI_result\Function_Analyse\Enrichment_Analyse\Pathway_Enrichment\AIH-VS-Normal_Pathway_enrichment\AIH-VS-Normal.htm#gene22) | 4 (2.34%) | 10 (0.77%) | 0.03239008 | ko05332 |
| **23** | [**Biosynthesis of unsaturated fatty acids**](file:///E:\001%20doge\%E6%B5%8B%E5%BA%8F-%E5%8D%8E%E5%A4%A7\%E7%BB%93%E6%9E%9C\report\BGI_result\Function_Analyse\Enrichment_Analyse\Pathway_Enrichment\AIH-VS-Normal_Pathway_enrichment\AIH-VS-Normal.htm#gene23) | 3 (1.75%) | 6 (0.46%) | 0.03340454 | ko01040 |
| **24** | [**Citrate cycle (TCA cycle)**](file:///E:\001%20doge\%E6%B5%8B%E5%BA%8F-%E5%8D%8E%E5%A4%A7\%E7%BB%93%E6%9E%9C\report\BGI_result\Function_Analyse\Enrichment_Analyse\Pathway_Enrichment\AIH-VS-Normal_Pathway_enrichment\AIH-VS-Normal.htm#gene24) | 3 (1.75%) | 6 (0.46%) | 0.03340454 | ko00020 |
| **25** | [**Autoimmune thyroid disease**](file:///E:\001%20doge\%E6%B5%8B%E5%BA%8F-%E5%8D%8E%E5%A4%A7\%E7%BB%93%E6%9E%9C\report\BGI_result\Function_Analyse\Enrichment_Analyse\Pathway_Enrichment\AIH-VS-Normal_Pathway_enrichment\AIH-VS-Normal.htm#gene25) | 10 (5.85%) | 41 (3.17%) | 0.03497068 | ko05320 |
| **26** | [**Viral myocarditis**](file:///E:\001%20doge\%E6%B5%8B%E5%BA%8F-%E5%8D%8E%E5%A4%A7\%E7%BB%93%E6%9E%9C\report\BGI_result\Function_Analyse\Enrichment_Analyse\Pathway_Enrichment\AIH-VS-Normal_Pathway_enrichment\AIH-VS-Normal.htm#gene26) | 11 (6.43%) | 47 (3.63%) | 0.03663714 | ko05416 |
| **27** | [**Linoleic acid metabolism**](file:///E:\001%20doge\%E6%B5%8B%E5%BA%8F-%E5%8D%8E%E5%A4%A7\%E7%BB%93%E6%9E%9C\report\BGI_result\Function_Analyse\Enrichment_Analyse\Pathway_Enrichment\AIH-VS-Normal_Pathway_enrichment\AIH-VS-Normal.htm#gene27) | 2 (1.17%) | 3 (0.23%) | 0.04750818 | ko00591 |
| **28** | [**Selenocompound metabolism**](file:///E:\001%20doge\%E6%B5%8B%E5%BA%8F-%E5%8D%8E%E5%A4%A7\%E7%BB%93%E6%9E%9C\report\BGI_result\Function_Analyse\Enrichment_Analyse\Pathway_Enrichment\AIH-VS-Normal_Pathway_enrichment\AIH-VS-Normal.htm#gene28) | 2 (1.17%) | 3 (0.23%) | 0.04750818 | ko00450 |
| **29** | [**Sulfur metabolism**](file:///E:\001%20doge\%E6%B5%8B%E5%BA%8F-%E5%8D%8E%E5%A4%A7\%E7%BB%93%E6%9E%9C\report\BGI_result\Function_Analyse\Enrichment_Analyse\Pathway_Enrichment\AIH-VS-Normal_Pathway_enrichment\AIH-VS-Normal.htm#gene29) | 2 (1.17%) | 3 (0.23%) | 0.04750818 | ko00920 |
| **30** | [**alpha-Linolenic acid metabolism**](file:///E:\001%20doge\%E6%B5%8B%E5%BA%8F-%E5%8D%8E%E5%A4%A7\%E7%BB%93%E6%9E%9C\report\BGI_result\Function_Analyse\Enrichment_Analyse\Pathway_Enrichment\AIH-VS-Normal_Pathway_enrichment\AIH-VS-Normal.htm#gene30) | 2 (1.17%) | 3 (0.23%) | 0.04750818 | ko00592 |
