# Supplementary material for: Plasma proteomic analysis of autoimmune hepatitis in an improved AIH mouse model
Source: J Transl Med. 2020 Jan 6;18:3. doi: 10.1186/s12967-019-02180-3 (PMC6943959; doi:10.1186/s12967-019-02180-3)

**Additional file 6: Figure S3** *A*) barplot of pathway analysis for all DEPs. *B*) The up- and down-regulated DEPs in the significant pathways. Red means up-regulated proteins and blue means down-regulated proteins. X-axis displays the name of pathway; y-axis displays the count of differentially expressed protein.


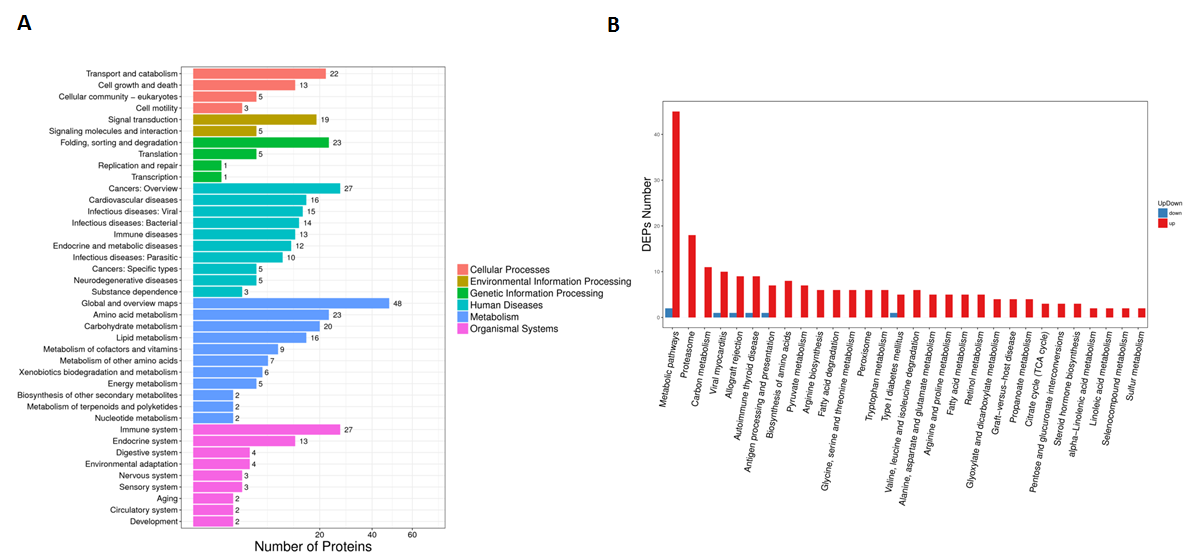

Supplement: Supplementary file 6 — Additional file 6: Figure S3.A) A barplot of pathway analysis for all DEPs. B) The up- and down-regulated DEPs in the significant pathways. Red means up-regulated proteins and blue means down-regulated proteins; X-axis displays the name of pathway; Y-axis displays the count of differentially expressed protein. [file 12967_2019_2180_MOESM6_ESM.docx]
