# Supplementary material for: Plasma proteomic analysis of autoimmune hepatitis in an improved AIH mouse model
Source: J Transl Med. 2020 Jan 6;18:3. doi: 10.1186/s12967-019-02180-3 (PMC6943959; doi:10.1186/s12967-019-02180-3)
Supplement: Supplementary file 7 — Additional file 7: Figure S4. The pathway map of antigen processing and presentation. Red means up-regulated and green means down-regulated DEPs. [file 12967_2019_2180_MOESM7_ESM.docx]

**Additional file 7: Figure S4** The pathway map of antigen processing and presentation. Red means up-regulated and green means down-regulated DEPs

**
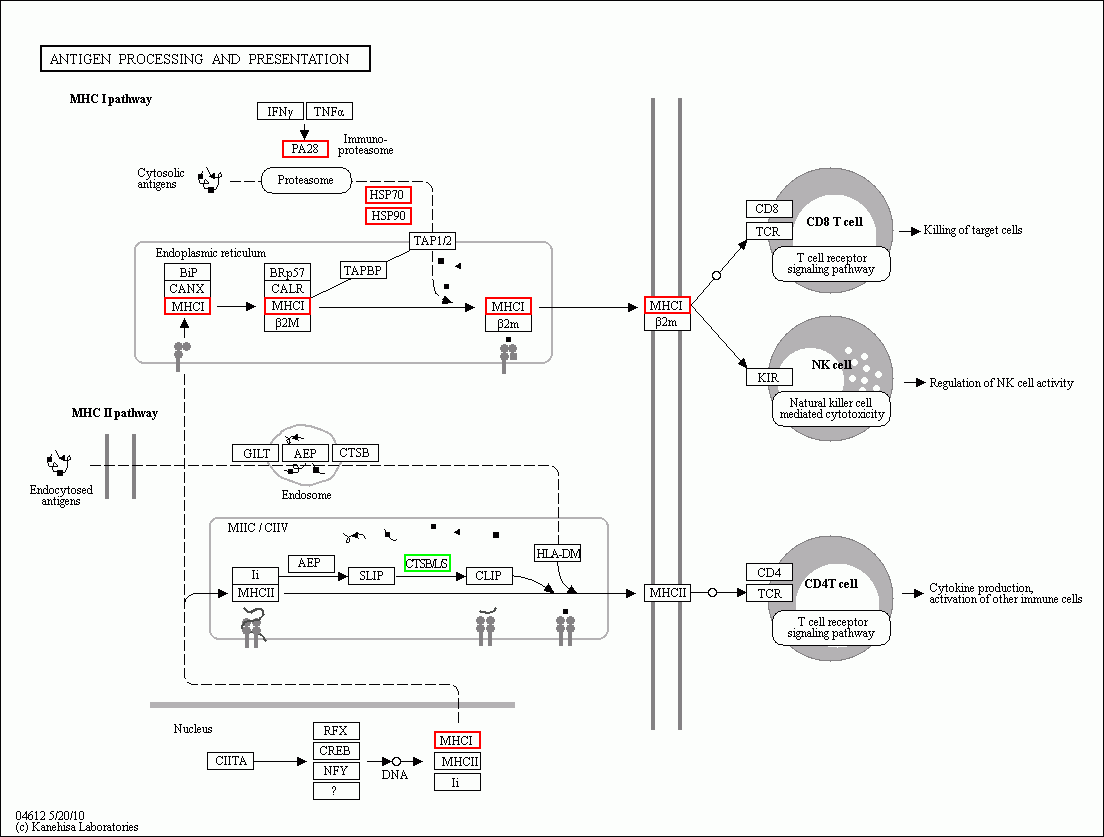
**
