# Supplementary material for: Plasma proteomic analysis of autoimmune hepatitis in an improved AIH mouse model
Source: J Transl Med. 2020 Jan 6;18:3. doi: 10.1186/s12967-019-02180-3 (PMC6943959; doi:10.1186/s12967-019-02180-3)
Supplement: Supplementary file 8 — Additional file 8: Table S6. Description and the mean ratio of the DEPs listed in Fig. 8. The comparison was conducted between the AIH mice group and normal mice group. Mean Ratio, SD, and P-value represent the statistical data of AIH mice vs. Normal mice by IBT (n = 5). [file 12967_2019_2180_MOESM8_ESM.docx]

**Additional file 8: Table S6** Description and the mean ratio of the DEPs listed in Figure 7. The comparison was conducted between the AIH mice group and normal mice group. Mean Ratio, SD, and P-value represent the statistical data of AIH mice vs. Normal mice by IBT (n=5).

| Protein_ID | Description | Protein_Coverage | Mean_Ratio | SD | P-value |
| --- | --- | --- | --- | --- | --- |
| sp\|P05366\|SAA1_MOUSE | Serum amyloid A-1 protein | 0.451 | 3.02 | 0.584 | 2.20E-16 |
| sp\|P05367\|SAA2_MOUSE | Serum amyloid A-2 protein | 0.525 | 2.78 | 0.665 | 2.20E-16 |
| sp\|P10810\|CD14_MOUSE | Monocyte differentiation antigen CD14 | 0.178 | 2.31 | 0.47 | 2.20E-16 |
| sp\|O35490\|BHMT1_MOUSE | Betaine--homocysteine S-methyltransferase 1 | 0.275 | 2.22 | 1 | 3.69E-09 |
| sp\|Q91WP6\|SPA3N_MOUSE | Serine protease inhibitor A3N | 0.49 | 2.13 | 0.92 | 1.26E-09 |
| sp\|P04918\|SAA3_MOUSE | Serum amyloid A-3 protein | 0.238 | 2.12 | 0.263 | 2.20E-16 |
| sp\|P63038\|CH60_MOUSE | 60 kDa heat shock protein, mitochondrial | 0.23 | 2 | 0.977 | 3.65E-07 |
| sp\|Q99KQ4\|NAMPT_MOUSE | Nicotinamide phosphoribosyltransferase | 0.031 | 2 | 0.683 | 7.74E-07 |
| sp\|Q64442\|DHSO_MOUSE | Sorbitol dehydrogenase | 0.269 | 1.98 | 0.647 | 8.74E-11 |
| sp\|Q61646\|HPT_MOUSE | Haptoglobin OS=Mus musculus | 0.389 | 1.94 | 0.75 | 4.68E-08 |
| sp\|P07901\|HS90A_MOUSE | Heat shock protein HSP 90-alpha | 0.317 | 1.81 | 0.702 | 7.64E-08 |
| sp\|P11087\|CO1A1_MOUSE | Collagen alpha-1(I) chain | 0.006 | 1.79 | 0.373 | 1.5E-11 |
| sp\|Q8K426\|RETNG_MOUSE | Resistin-like gamma | 0.128 | 1.77 | 0.495 | 5.89E-08 |
| sp\|P16014\|SCG1_MOUSE | Secretogranin-1 OS=Mus musculus | 0.01 | 1.71 | 0.713 | 6.64E-06 |
| sp\|P16014\|SCG1_MOUSE | Secretogranin-1 | 0.01 | 1.71 | 0.713 | 6.64E-06 |
| sp\|P28843\|DPP4_MOUSE | Dipeptidyl peptidase 4 | 0.011 | 1.69 | 0.545 | 3.52E-07 |
| sp\|Q8VCM7\|FIBG_MOUSE | Fibrinogen gamma chain OS=Mus musculus | 0.106 | 1.66 | 0.774 | 6.25E-05 |
| sp\|Q9JHH6\|CBPB2_MOUSE | Carboxypeptidase B2 OS=Mus musculus | 0.18 | 1.65 | 0.452 | 2.89E-08 |
| sp\|P28798\|GRN_MOUSE | Granulins OS=Mus musculus | 0.017 | 1.63 | 0.444 | 8.21E-09 |
| sp\|Q61316\|HSP74_MOUSE | Heat shock 70 kDa protein 4 | 0.197 | 1.63 | 0.348 | 1.17E-10 |
| sp\|P28798\|GRN_MOUSE | Granulins | 0.017 | 1.63 | 0.444 | 8.21E-09 |
| sp\|P51859\|HDGF_MOUSE | Hepatoma-derived growth factor | 0.038 | 1.63 | 0.654 | 2.63E-06 |
| sp\|Q8R1L8\|ANGL8_MOUSE | Angiopoietin-like protein 8 | 0.076 | 1.56 | 0.306 | 2.25E-10 |
| sp\|Q7M747\|S2B24_MOUSE | Secretoglobin family 2B member 24 | 0.062 | 1.54 | 0.573 | 8.04E-05 |
| sp\|Q6P069\|SORCN_MOUSE | Sorcin OS=Mus musculus | 0.212 | 1.51 | 0.274 | 1.44E-11 |
| sp\|P12399\|CTL2A_MOUSE | Protein CTLA-2-alpha | 0.058 | 0.65 | 0.101 | 1.34E-13 |
| sp\|Q8CGD2\|CRLD1_MOUSE | Cysteine-rich secretory protein LCCL domain-containing 1 | 0.024 | 0.65 | 0.215 | 9.87E-08 |
| sp\|P11589\|MUP2_MOUSE | Major urinary protein 2 | 0.244 | 0.63 | 0.193 | 9.66E-09 |
| sp\|Q6PHQ8\|NAA35_MOUSE | N-alpha-acetyltransferase 35, NatC auxiliary subunit | 0.014 | 0.62 | 0.138 | 4.15E-10 |
| sp\|Q19LI2\|A1BG_MOUSE | Alpha-1B-glycoprotein | 0.305 | 0.58 | 0.235 | 2.00E-07 |
| sp\|P47876\|IBP1_MOUSE | Insulin-like growth factor-binding protein 1 | 0.026 | 0.53 | 0.117 | 2.92E-13 |
